# Insight from Carbon and Nitrogen Isotope Fractionation of Sulfamethoxazole during Oxidative vs Reductive Transformation

**DOI:** 10.1021/acs.est.6c07718

**Published:** 2026-10-01

**Authors:** Aoife Canavan, Christopher Dirr, Martin Elsner

**Affiliations:** Chair of Analytical Chemistry and Water Chemistry, Department of Chemistry, TUM School of Natural Sciences, Technical University of Munich, Lichtenbergstraße 4, 85748 Garching, Germany

**Keywords:** sulfamethoxazole, abiotic transformation, oxidation, reduction, isotope fractionation

## Abstract

The antibiotic sulfamethoxazole (SMX) is frequently detected in aquatic systems, raising environmental concerns. Therefore, it is necessary to investigate its transformation pathways under diverse environmental conditions. Here, we explored information from stable isotope fractionation during reductive transformation of SMX with Fe­(II)-amended goethite suspensions vs oxidative transformation with MnO_2_. Transformation of SMX by surface-bound Fe­(II) showed significant carbon isotope fractionation (ε_C_ = −3.6‰ ± 0.1‰ and ε_C_ = −2.9‰ ± 0.2‰, with and without buffer, respectively), pronounced nitrogen isotope fractionation (ε_N_ = −12.8‰ ± 0.6‰ and ε_N_ = −13.0‰ ± 0.2‰, with and without buffer, respectively), and evidence of transformation products that are consistent with initial reductive N–O bond cleavage. In contrast, reaction with MnO_2_ caused normal carbon but inverse nitrogen isotope fractionation (ε_C_ = −1.7‰ ± 0.1‰ and ε_C_ = −2.7‰ ± 0.2‰, with and without buffer, respectively, ε_N_ = +1.9‰ ± 0.2‰ without buffer) indicative of an initial oxidative electron transfer. Oxidation products were not easily identified by high-resolution mass spectrometry, emphasizing the importance of compound-specific isotope analysis (CSIA) as an alternative for detecting ongoing transformation. Our study thus highlights the potential of CSIA to detect transformation in settings where concentration and product analysis would be inconclusive and to distinguish between reductive and oxidative transformation pathways of SMX in the environment.

## Graphical abstract

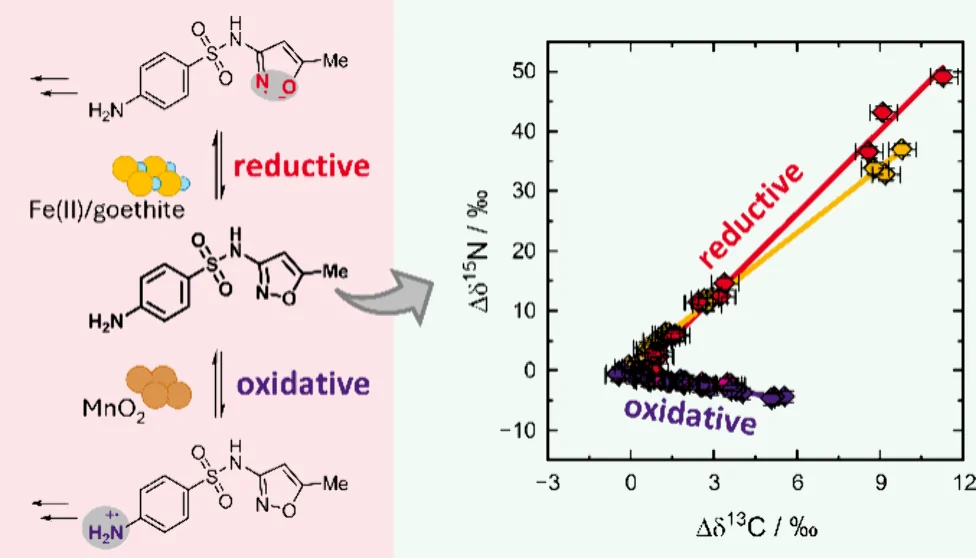

## Introduction

The presence of antibiotics in the environment raises concerns about human health, as residual concentrations may promote antibiotic resistance.^1^ Among them, sulfamethoxazole (SMX) stands out, since it is frequently detected in aquatic and terrestrial environments.^2–4^ SMX is widely used in human and veterinary medicine and enters surface waters through wastewater effluents, agricultural runoffs, and improper disposal.^5,6^ The use of the antibiotic in livestock farming and the application of animal manure in agriculture can release SMX into the soil, potentially entering food webs and posing a risk to human health.^1,7,8^ There, it is not immediately transformed, underscoring the need to develop a comprehensive understanding of SMX’s environmental behavior, fate, and underlying transformation pathways.^9^ Transformation of SMX includes biotic transformation by microorganisms or plants and abiotic pathways involving chemical reactions, such as photolysis or reductive transformation.^10^ Microbial transformation of SMX has been characterized to occur under both aerobic and anaerobic conditions, resulting in reactions such as acetylation,^11^ ipso-hydroxylation,^12^ as well as reductive N–O bond cleavage.^13^ Phototransformation can give rise to multiple bond cleavages, leading to the formation of various transformation products (TPs), including sulfanilic acid and sulfanilamide.^14^ Abiotic reactions between SMX and Fe­(III) mineral-associated Fe­(II) resulted in reduced and isomerized forms of the isoxazole moiety in SMX.^15^ Conventional methods for evaluating the environmental fate of SMX rely on monitoring changes in concentration and detecting TPs. However, nondegradative processes such as sorption, volatilization, and dilution can also affect SMX concentrations in the environment, and distinguishing these processes from transformation is difficult. Assessments may be biased if the parent compound is repeatedly reintroduced, and TPs may remain undetected if they are further transformed. Therefore, transformation - and associated transformation pathways - may not be adequately assessed, particularly in field conditions where multiple pathways often occur simultaneously.^16^

In this context, compound-specific isotope analysis (CSIA) has been shown to offer new prospects for assessing the transformation of environmental contaminants, even when TPs are unknown.^17–19^ This complementary approach relies on measuring stable isotope ratios (e.g., ^13^C/^12^C or ^15^N/^14^N) of the parent compound at natural abundance. On the one hand, these isotope ratios may be indicative of different sources of the same chemical. CSIA allows determination of the origin of contaminants based on their stable isotope ratios, as these ratios depend on the raw materials, production pathways, and environmental processes associated with their sources.^20^ CSIA has shown that it can differentiate SMX from various manufacturers based on carbon or nitrogen isotope ratios.^21,22^ On the other hand, changes in this isotope ratio (“isotope fractionation”) may be observable if the parent compound is transformed. These changes are caused by kinetic isotope effects at reactive positions, because light and heavy isotopes of an element (e.g., ^12^C and ^13^C) have slightly different activation energies during bond cleavage or formation. Therefore, specific transformation reactions can result in distinct isotope effects in the parent compound, providing independent evidence of contaminant transformation and complementary insight into reaction mechanisms to that from product formation.^23,24^ Analyzing isotope fractionation across multiple elements (e.g., N, S, and/or H in addition to C) provides another level of certainty in elucidating transformation mechanisms compared to looking at only one element. This is because isotope fractionation of a single element may be masked if a rate-determining, nonfractionating step (e.g., mass transfer) precedes the isotope-sensitive bond cleavage. However, such kinetic limitations influence fractionation of all elements similarly, so that dual-element isotope fractionation relationships are much less affected.^24^ Recently, much attention has been directed at isotope fractionation of SMX transformation in natural and engineered systems. Early studies of SMX transformation examined only carbon isotope fractionation during aerobic microbial transformation,^25^ photolysis,^25,26^ and oxidation by ozone or chlorine dioxide.^27^ Later methodological advances enabled dual- and multi-element CSIA, providing isotope data for anaerobic microbial degradation,^28^ phototransformation,^29^ and heat-activated persulfate oxidation,^30^ underscoring its potential to improve pathway discrimination. Nevertheless, a clear and systematic distinction between reductive and oxidative SMX transformation processes has not yet been established, demonstrating the need for further multi-element isotope studies.

Although several transformation pathways of SMX have been studied by CSIA, abiotic transformation pathways with naturally occurring minerals have not yet been addressed but may also play a key role. Oxidative transformation by MnO_2_ and reductive transformation by surface-bound Fe­(II) are arguably the most important abiotic transformation pathways in natural systems. Manganese oxides are among the strongest naturally occurring oxidants in soils and sediments and are a key driver of organic compound transformation, including a wide range of organic contaminants, such as aromatic amines.^31–36^ In anoxic environments, in turn, Fe­(II) plays a crucial role in biogeochemical processes. It can interact with Fe­(III)-bearing minerals, involving electron transfer and redox processes that affect the minerals’ reactivity.^37–39^ Mineral-bound Fe­(II) has been shown to reduce chlorinated hydrocarbons and nitroaromatic compounds in various field and laboratory studies.^40–43^ Rates of contaminant transformation varied by several orders of magnitude depending on the Fe­(III)-bearing mineral that served as a sorbent for Fe­(II), the amount of aqueous Fe­(II), and the pH.^44–46^ In laboratory studies, the pH is commonly maintained using buffers, such as zwitterionic sulfonate buffers, including 3-morpholinopropane-1-sulfonic acid (MOPS). However, their presence may also interfere with the surface chemistry of goethite, thereby affecting reaction kinetics and the distribution of TPs, as shown for the reduction of tetrachloromethane.^47^ Since isotope fractionation can reflect different product formation pathways, this parameter also needs to be investigated.

The aims of this study were, therefore, (i) to explore the nature and magnitude of carbon and nitrogen isotope fractionation that can be observed during SMX transformation using Fe­(II)-amended goethite and MnO_2_ suspensions, (ii) to determine whether these environmentally important abiotic transformation reactions may be distinguished by characteristically different trends of carbon and nitrogen isotope fractionation that is observable in the form of different slopes in dual-element isotope plots, (iii) to evaluate whether the observed isotope effects can provide mechanistic insight into the reactions in the two model systems, (iv) to investigate whether the presence of buffer influences the observed isotope effects during the reductive transformation of SMX by surface-bound Fe­(II), and (v) if so, to link them to differences in the underlying reaction mechanism.

## Materials and Methods

### Chemicals and Solvents

A detailed description of the analytical standards, chemicals, and solvents used, including their purities and suppliers, is provided in the Supporting Information (S1). Fully ^13^C-labeled SMX was synthesized following the procedure of Canavan et al.,^48^ using the respective isotopically labeled starting materials (S2). Singly ^13^C- and ^15^N-labeled SMX were obtained as previously reported by Canavan et al.^48^

### Working under Anoxic Conditions

Anoxic experiments were performed inside a glovebox (nitrogen atmosphere, oxygen <0.5 ppm), and the glassware used was placed in the glovebox 3 days prior to use. For the anoxic experiments, ultrapure water was degassed by heating to 60 °C and sparging with argon for 3 h while vigorously stirring.

### Preparation of Solutions and Suspensions Used in Transformation Experiments

#### Preparation of an Fe­(II) Solution

An Fe­(II) stock solution was prepared by adding hydrochloric acid (37 wt %, 5.17 mL) and iron powder (1.82 g) to freshly degassed water (50 mL). The reaction mixture was subsequently stirred for 2 h at 80 °C. The light blue, gray suspension was filtered through a syringe filter (25 mm PTFE syringe filter, hydrophilic, 0.22 μm, BGB) inside the glovebox, resulting in a light blue solution.

#### Preparation of a MnO_2_ Suspension

The MnO_2_ synthesis was performed according to the procedure described by Murray.^49^ Briefly, a NaMnO_4_ solution (4 M, 305 μL, 1.2 mmol, 1.0 equiv) was diluted with ultrapure water (300 mL) and purged with argon for 30 min while stirring. After the addition of a sodium hydroxide solution (1 M, 2.4 mL), a MnCl_2_ solution (354 mM, 1.77 mmol, 350 mg, 1.5 equiv) was added dropwise to the purple reaction solution, while still purging with argon. A brown precipitate formed during the addition, and the reaction was stirred for additional 2 h at room temperature. The brown precipitate was washed with water until the conductivity fell below 3 μS cm^–1^. The concentration of the final MnO_2_ stock suspension was determined gravimetrically by evaporation of 4 mL of the suspension under a gentle stream of nitrogen (1.5 L min^–1^, 40 °C, TurboVap, Biotage, Sweden) until complete dryness. The dried MnO_2_ particles were characterized by powder XRD (S3 and Figure S1).

### Transformation by Fe­(II)-Amended Goethite Suspensions

The transformation of SMX with Fe­(II) and goethite was conducted in 1 L glass bottles under anoxic conditions in triplicate, according to Mohatt et al.^15^ The surface area of the goethite used was characterized according to the BET method (S3). The final concentrations of the reactants were 8 g L^–1^ goethite, 0.5 mM Fe­(II), 0.2 M NaCl, and 50 μM SMX at pH 7.0 ± 0.2. The pH was adjusted throughout the experiment by manually adding NaOH (1 M) or maintained using 50 mM MOPS, resulting in a pH of 7.0 ± 0.2. For the experiments without a buffer, the pH was adjusted every 2 min for the first 30 min, then every 30 min thereafter. As a result, the pH never dropped below 6.8. The ultrapure water or buffer, goethite, and Fe­(II) solution were mixed, and the suspension’s pH was adjusted to 7.0 ± 0.2 with 1 M hydrochloric acid and 1 M sodium hydroxide. After stirring the suspensions overnight to equilibrate the Fe­(II)/goethite system, the reaction was initiated by adding an SMX solution (0.25 mM, 220 mL). The reaction suspension was continuously stirred in the dark. At certain time points, different amounts of the suspension were quenched by adding sodium hydroxide as pellets or as a solution (1 M) inside the glovebox, resulting in a pH of approximately 12, followed by immediate filtration (25 mm PTFE, hydrophilic, 0.22 μm, BGB) outside the glovebox.^50^ Afterward, 1 mL was used for concentration analysis, and the remaining sample was concentrated by solid-phase extraction for stable isotope analysis. Simultaneously, a fourth experiment for each condition was conducted, from which 1 mL samples were taken and directly quenched inside the glovebox by filtration (25 mm PTFE, hydrophilic, 0.22 μm, BGB). Control experiments without goethite or Fe­(II) in MOPS buffer at pH 7.0 ± 0.2 showed no transformation during the sampling period (Figure S2a). Quenching by filtration through a syringe filter or by adding sodium hydroxide to reach pH 12, followed by filtration through a syringe filter, showed no difference (Figure S2a).

### Transformation by MnO_2_

The transformation of SMX by MnO_2_ was carried out in 1 L or 500 mL glass bottles in triplicate at pH 3.0 ± 0.2. To this end, the final suspensions consisted of 21 mg L^–1^ MnO_2_ and 100 μM SMX at pH 3.0 ± 0.2. The pH was adjusted throughout the experiment by manually adding HCl (1 M) or maintained using 10 mM phosphate buffer, resulting in a pH of 3.0 ± 0.2. For the experiments without a buffer, the pH was adjusted every hour for the first 8 h, followed by daily adjustments. As of day 3, no further adjustments were necessary, and as a result, the pH never rose above 3.2. After adding the MnO_2_ stock suspension, the pH was adjusted to 3.0, and the suspensions were stirred for 1.5 h to equilibrate. An aqueous SMX solution (400 μM, 125 mL) was added to initiate each transformation reaction. For concentration analysis, 1 mL of the suspension was quenched with oxalic acid (0.5 M, 150 μL) at selected time points, which led to dissolution of the manganese oxide. Samples for isotope analysis were taken in volumes ranging from 13 to 133 mL, quenched by filtration through a syringe filter (25 mm PTFE, hydrophilic, 0.22 μm, BGB), and concentrated by solid-phase extraction for stable isotope analysis. Control experiments showed no transformation in phosphate buffer at pH 3.0 ± 0.2 (Figure S2b). Quenching by filtration through a syringe filter (dissolved SMX) or by the addition of oxalic acid (dissolved and sorbed SMX) showed no difference (Figure S2b).

#### Experiments for Transformation Product Identification

To elucidate the structures of TPs, additional experiments were performed with entirely ^13^C-labeled SMX and SMX labeled at a single carbon or nitrogen atom, respectively. These experiments were conducted under identical reaction conditions and initial concentrations to those used for SMX at natural abundance, as described above, with the sole modification that a reaction volume of 100 mL was used. Each reaction was quenched after 8 days by filtration through a syringe filter or by the addition of oxalic acid, yielding two sets of samples quenched by two methods. Nonpurgeable organic carbon was analyzed using TOC-L (Shimadzu, Japan) for two filtered samples from day 0 to day 8. Qualitative analysis of CO_2_ formation was performed by conducting an experiment with entirely labeled SMX in 20 mL crimp vials and tracing the labeled ^13^CO_2_ in the headspace. To this end, 1 mg of ^13^C_10_-labeled SMX was added to the vial, which was then tightly sealed with a rubber septum and purged with helium. The reaction was initiated by adding 2 mL of a MnO_2_ suspension at pH 3.0, which was then stirred for 14 days.

### Solid-Phase Extraction of Sulfamethoxazole

The pH of the Fe­(II)/goethite samples was adjusted to 3.7, whereas the pH of the samples from the MnO_2_ experiments was not changed, and solid-phase extraction (SPE) was performed using Oasis HLB cartridges (6 mL, 200 mg; Waters, Milford, MA, USA) according to Liu et al.^29^ The cartridges were preconditioned by dropwise percolation with acetonitrile (5 mL), followed by ethyl acetate (5 mL), and then water (5 mL). Next, the samples were dropwise percolated through the cartridges and dried under vacuum overnight. The SMX was eluted from the cartridges with ethyl acetate (5 mL) followed by acetonitrile (5 mL). The eluate was dried under a gentle stream of nitrogen (0.5 L min^–1^, 25 °C, TurboVap, Biotage, Sweden) to complete dryness and reconstituted in methanol (0.5 or 1 mL).

### Concentration Analysis by HPLC-UV/vis

Concentration analysis of SMX was performed on a Nexera XR HPLC system, consisting of two solvent pumps (LC-20AD), an autosampler (SIL-20AC XR), a column oven (CTO-20A prominence), and a diode array detector (SPD-M20A prominence) (all, Shimadzu, Japan). SMX was separated on a RP18 column (XTerra RP18 Column, length: 150 mm, diameter: 3.0 mm, particle size: 3.5 μm, Waters, USA) at 40 °C with a flow rate of 0.5 mL min^–1^ using a binary gradient with mobile phase A (water with 0.1% formic acid for MnO_2_ transformation samples and water with 0.5% formic acid for Fe­(II)/goethite transformation samples) and mobile phase B (methanol). Eluent B was held at 10% for 1 min, then increased to 90% over the next 8 min, held for 1 min, and then returned to 10% over 2 min. External calibration was used to quantify SMX concentrations in samples during the transformation reactions using a wavelength of 268 nm. Data processing was performed by LabSolutions, version 5.117 (Shimadzu, Japan).

### Stable Isotope Analysis by GC-IRMS

#### Carbon Isotope Analysis

Analysis was performed with a method adapted from Ouyang et al.^28^ on a Trace 1310 gas chromatograph connected via a GC IsoLink II and a ConFlo IV interface to a Finnigan MAT 253 isotope ratio mass spectrometer (all, Thermo Fisher Scientific, Germany). To this end, 1.5 μL of the samples was injected into a split/splitless injector equipped with a splitless liner (4 mm ID × 78.5 mm length, 800 μL, ultra inert, double taper, without glass wool, Agilent Technologies, USA) using a TriPlusRSH (Thermo Fisher Scientific, Germany). The injector was operated in split mode with a split ratio of 1:3 at 280 °C. For the separation of the target analyte, an Agilent DB-1 column (30 m × 0.32 mm × 1.00 μm, Agilent Technologies, USA) with pre- and postcolumn (each 1 m deactivated fused silica guard column, 0.32 mm ID, Agilent, USA) with a constant helium flow rate of 2.0 mL min^–1^ was used with a GC-oven starting temperature of 170 °C, increasing to 320 °C with a ramp of 20 °C min^–1^ and a hold time of 5 min. After elution, the eluates were oxidized at 1000 °C in a commercial combustion reactor (NiO tube CuO-NiO reactor, 2 mm, Thermo Fisher, Germany). The reactor was initially preoxidized by flushing with oxygen for 480 min at 600 °C, 240 min at 900 °C, and 120 min at 1000 °C. Between each step, the reactor was vented for 1 min, followed by keeping it in backflush mode for 1 min, and after the final oxidation step, the system was kept in backflush mode for 60 min. The reactor was oxidized for 15 s before each injection to ensure consistent oxidation conditions, followed by a 2 min backflush. The limit of precise isotope analysis was determined using varying SMX concentrations (2–20 nmol of C on column) of an in-house SMX standard (Figure S3a). Standards and samples were measured in triplicate, while monitoring retention times and isotope values for an in-house standard of SMX and USGS62 (caffeine) used for bracketing samples ensured system stability and measurement reproducibility. The software Isodat 3.0 (Thermo Fisher Scientific, Germany) automatically performed peak detection and integration, using the individual background algorithm for baseline correction.

#### Nitrogen Isotope Analysis

Before GC-IRMS measurements for nitrogen stable isotope analysis, SMX was derivatized according to Dou and Canavan et al.^22^ Briefly, 900 μL of methanol and 100 μL of an SMX extract were added to a 20 mL crimp cap vial. Next, 160 μL of trimethylsilyl diazomethane (1.8–2.4 M in hexanes) was added, and the vial was tightly closed. After placing the vial in a water bath at 50 °C for 1 h, the solvent was evaporated under a gentle stream of nitrogen (0.5 mL min^–1^, 25 °C, TurboVap, Biotage, Sweden) and the residue was reconstituted in methanol (267 μL for Fe­(II)/goethite; 237 μL for MnO_2_). The stable nitrogen isotope analysis was performed on a GC-IRMS consisting of a Trace GC Ultra connected via a Finnigan GC Combustion III interface to a Finnigan MAT 253 mass spectrometer (all, Thermo Fisher Scientific, Germany). The system was equipped with a customized oxidation reactor consisting of an alumina tube (320 mm length, 0.5 mm ID, 1.5 mm OD, Elemtex, U.K.) enclosing two nickel wires (0.1 mm diameter, 99.994% purity, Alfa Aesar, Germany) and one platinum wire (0.1 mm diameter, 99.997% purity, Thermo Fisher Scientific, Germany), and a customized reduction reactor filled with three copper wires (0.1 mm diameter, 99.9999% purity, Alfa Aeser, Germany). Therefore, 3 μL of the derivatized sample was injected into a split/splitless injector equipped with a splitless liner (5 mm ID × 106 mm length, Agilent, USA) maintained at 250 °C using a GC PAL autosampler (CTC Analytics AG, Switzerland). Splitless mode was used for the first minute at a surge pressure of 250 kPa and afterward switched to split mode with a split flow rate of 20 mL min^–1^. An Agilent J&W DB-5MS UI column (30 m length × 0.25 mm ID × 1.0 μm film thickness, Agilent Technologies, USA) with pre- and postcolumn (each 1 m deactivated fused silica guard column, 0.25 mm ID, Agilent, USA) with a constant helium flow rate of 1.4 mL min^–1^ was used for the separation of the compounds. The GC oven was initially held at 120 °C for 1 min, then ramped to 250 °C at 22 °C min^–1^, and finally ramped to 325 °C at 40 °C min^–1^, where it was held for 9 min. Samples containing a high amount of coenriched TPs (last samples of Fe­(II)/goethite experiments) were separated on an Agilent J&W DB-5MS UI column (60 m length × 0.25 mm ID × 1.0 μm film thickness, Agilent Technologies, USA) while the GC-oven was held at 120 °C for 1 min, increased to 250 °C with a ramp of 40 °C min^–1^ and then further increased to 325 °C with a ramp of 40 °C min^–1^, where it was held for 15 min. After GC elution, the compound peaks were oxidized at 1030 °C, followed by reduction at 650 °C to remove unwanted nitrogen oxides. To ensure consistent oxidation, the reactor was oxidized for 20 min and subsequently held in backflush for 20 min to remove any undesired oxidation products before each injection. A liquid nitrogen trap preceded the entry of the gases into the IRMS to capture the CO_2_ generated during combustion. This ensured that CO_2_ did not enter the ion source, preventing any potential interference with the measurement of N_2_ isotopologues. The trap was emptied daily by removing the liquid N_2_ during reoxidation of the reactor. The limit of precise nitrogen isotope analysis was determined by derivatizing varying SMX concentrations (4–16 nmol of N on column) of an in-house SMX standard (Figure S3b). Standards and samples were measured in duplicate, while monitoring retention times and isotope values of a freshly derivatized in-house SMX standard used for bracketing samples ensured system stability and measurement reproducibility. The software Isodat 3.0 (Thermo Fisher Scientific, Germany) automatically performed peak detection and integration, using the individual background algorithm for baseline correction.

### Transformation Product Analysis by LC-Orbitrap

Separation of the two major TP peaks of the reduction experiments was performed on the same HPLC device used for quantifying residual SMX, including a fraction collector (FRC-10A, Shimadzu, Japan). Separation of the TPs was achieved with a biphenyl column (Kinetex biphenyl column, length 100 mm, diameter 4.6 mm, particle size 5 μm, Phenomenex, USA) using a binary gradient with mobile phase A (water with 0.5% formic acid) and mobile phase B (methanol). Eluent B was kept at 10% for the first 5 min, then increased to 50% over the next 10 min, held for 3 min, and then reduced to the starting percentage of 10% over the next 2 min. Fractions containing the two major TPs were collected and used for structural identification. To this end, the 40–80 μL from the collected fractions were directly infused at a flow rate of 4 μL min^–1^ using an autosampler (HT VH-A10-A) into an Orbitrap Exploris 240 equipped with an atmospheric pressure ion source OptaMax NG using a nonheated electrospray ionization probe equipped with a low-flow needle insert (all, Thermo Fisher Scientific, Germany). The system operated in positive mode. After identifying the *m*/*z* of the respective TP, its *m*/*z* was selected with a mass window of the quadrupole of 5 *m*/*z*, and the energy of the ion routing multipole was increased by 5 V every 2 min, resulting in distinct fragments of the respective TPs. The minor TPs of both transformation experiments were identified using a Vanquish liquid chromatography system coupled to an electrospray ionization Orbitrap Exploris 240 mass spectrometer (both Thermo Fisher Scientific, Germany). The Vanquish liquid chromatography system consisted of a split sampler HT VH-A10-A, binary pump H VH-P10-A, diode array detector (DAD) FG VF-D11-A, and column compartment VH-C10-A (all, Thermo Fisher Scientific, Germany). Separation of the TPs was achieved with a biphenyl column (Kinetex biphenyl column, length 150 mm, diameter 4.6 mm, particle size 5 μm, Phenomenex, USA) using a binary gradient with mobile phase A (water with 0.1% formic acid) and mobile phase B (water/methanol 9:1 with 0.1% formic acid). Eluent B was held at 11.1% for the first 5 min, then increased to 55.6% over the next 10 min, further increased to 100% B over 23 min, and held for 2 min at 100% B before returning to 11.1% B. An injection volume of 20–50 μL was used. For ionization, the atmospheric pressure ion source OptaMax NG was used with an H-ESI probe equipped with a high-flow needle insert (Thermo Fisher Scientific, Germany). Positive and negative ionization modes were used to analyze the TPs. The ESI spray was optimized before each sequence to ensure a stable, intense TIC (relative standard deviation of <5%) by adjusting the spray voltage, sheath gas, auxiliary gas, and sweep gas. Details on the ion source settings, scan properties, and MS^2^ scan properties are provided in Table S2. The TPs were selected by target mass and fragmented using a higher energy collisional dissociation (HCD) cell for MS^2^ analysis. For structure elucidation, the resulting mass spectra were compared with the mzCloud database, molecular formulas were predicted in FreeStyle (version 1.8.65.0), and the identification confidence was evaluated according to Schymanski et al.^51^

#### Structure Elucidation of Transformation Products Using Isotopically Labeled Sulfamethoxazole

For MnO_2_ transformation experiments, samples after 7 days were compared, where (i) uniformly ^13^C-labeled, (ii) position-specific ^13^C- and ^15^N-labeled, and (iii) SMX at natural abundance were used. Using LC-MS/MS data from these four experiments, TPs were characterized by comparing chromatographic retention times and respective fragmentation patterns. The uniformly ^13^C-labeled SMX enabled the determination of the number of carbon atoms retained in a TP based on characteristic mass shifts relative to the TP of the nonlabeled compound. Complementarily, position-specific isotope labels allowed the tracing of individual carbon and nitrogen atoms through both precursor and fragment ions, providing direct evidence of the preservation or loss of specific molecular moieties during transformation.

### Qualitative Detection of ^13^CO_2_ Formation by GC-IRMS

Analysis was performed on the same instrument as the carbon isotope analysis of SMX, with the following modifications. From the 20 mL crimp vial containing the reaction solution, 50 μL from the headspace was manually injected. The injector was operated in split mode with a split ratio of 1:6 at 240 °C. For chromatographic separation of CO_2,_ two Agilent GS-Q columns (each 30 m × 0.32 mm, Agilent Technologies, USA) with pre- and postcolumn (each 1 m guard column, 0.32 mm ID, Agilent, USA) with a constant helium flow rate of 2 mL min^–1^ were used with a GC-oven temperature of 60 °C.

### Data Evaluation

#### Isotope Values and Isotope Fractionation

All isotope values are reported in per mil (‰) as arithmetic means of triplicate or duplicate measurements with associated 95% confidence intervals (CIs, 0.5‰ for carbon and 1‰ for nitrogen isotope values) relative to the international reference material Vienna PeeDee Belemnite (VPDB) for carbon and air for nitrogen isotope analysis (eqs 1 and 2).

1 δC13=(C13/C12)sample−(C13/C12)reference(C13/C12)reference

2 δN15=(N15/N14)sample−(N15/N14)reference(N15/N14)reference

Isotope fractionation ε of carbon and nitrogen was calculated, following the Rayleigh equation (eq 3).

3 $$\frac{1+\delta_{t}}{1+\delta_{0}}={\left(\frac{C_{t}}{C_{0}}\right)}^{\varepsilon }$$

Here, δ is the isotopic composition of carbon (δ^13^C) or nitrogen (δ^15^N), and *c*_*t*_/*c*_0_ is the remaining fraction of SMX, while *t* represents the sampling time with 0 denoting the initial time before the reaction occurred. The isotopic fractionation of carbon (ε_c_) and nitrogen (ε_N_) was determined by the slope of the linear regression after linearization of eq 3. The errors of ε_c_ and ε_N_ values were reported as 95% CIs determined by regression analysis. Dual-element isotope slopes (Λ_N/C_) were calculated by the York method according to Ojeda et al.,^52^ using the IsoPlot R package (version 6.8) (eq 4).

4 ΛN/C=ΔδN15ΔδC13≈ε15Nε13C

The quality of the fit of York-regression slopes was evaluated using the mean square of weighted deviates (MSWD) and *p*-values. The uncertainties of the Λ_N/C_ values were given as the respective standard error of the slope.

#### Reaction Kinetics

For first-order kinetics, the time dependence of residual SMX is described by the following equation (eq 5).

5 $$c_{t}=c_{0}\;\exp\left(-kt\right)$$

Here, *c*_*t*_ is the SMX concentration at time *t*, *c*_0_ is the initial concentration, and *k* is the first-order rate constant. The associated uncertainty in the rate constant is reported as 95% CI.

## Results and Discussion

### Carbon and Nitrogen Isotope Fractionation during Abiotic Transformation of SMX

SMX was transformed by surface-bound Fe­(II) in the presence and absence of MOPS buffer, showing significant carbon isotope fractionation (ε_C_ = −3.6‰ ± 0.1‰ and ε_C_ = −2.9‰ ± 0.2‰ in the presence and absence of buffer, respectively; Figure 1a), as well as pronounced nitrogen isotope fractionation (ε_N_ = −12.8‰ ± 0.6‰ and ε_N_ = −13.0‰ ± 0.2‰, in the presence and absence of buffer, respectively; Figure 1b). For both elements, a normal isotope effect was observed, meaning that SMX molecules with light isotopes (^12^C and ^14^N) reacted faster, leaving the molecules with heavy isotopes (^13^C and ^15^N) in the residual substrate. As a result, the ratios of ^13^C to ^12^C and of ^15^N to ^14^N increased over time. In contrast to the transformation by surface-bound Fe­(II), transformation by MnO_2_ caused small inverse nitrogen isotope fractionation (ε_N_ = +1.9‰ ± 0.2‰ in the absence of buffer; Figure 1d), meaning that ^15^N-containing molecules reacted faster and the ratios of ^15^N to ^14^N in the remaining SMX decreased over time. Also in the buffered system, a subtle trend toward an inverse isotope effect was found (δ^15^N_*t*=0_ = 0.7‰ ± 1‰ versus δ^15^N_*t*=42d_ = −0.9‰ ± 1‰). For carbon, again, a small normal isotope effect was observed (ε_C_ = −1.7‰ ± 0.1‰ and ε_C_ = −2.7‰ ± 0.2‰, in the presence and absence of buffer, respectively; Figure 1c). Hence, isotope values of carbon and nitrogen changed as the amount of SMX decreased (Figure 1a–d), with the two processes exhibiting distinct trends for each element. This will be discussed in the following.

**Figure 1:**
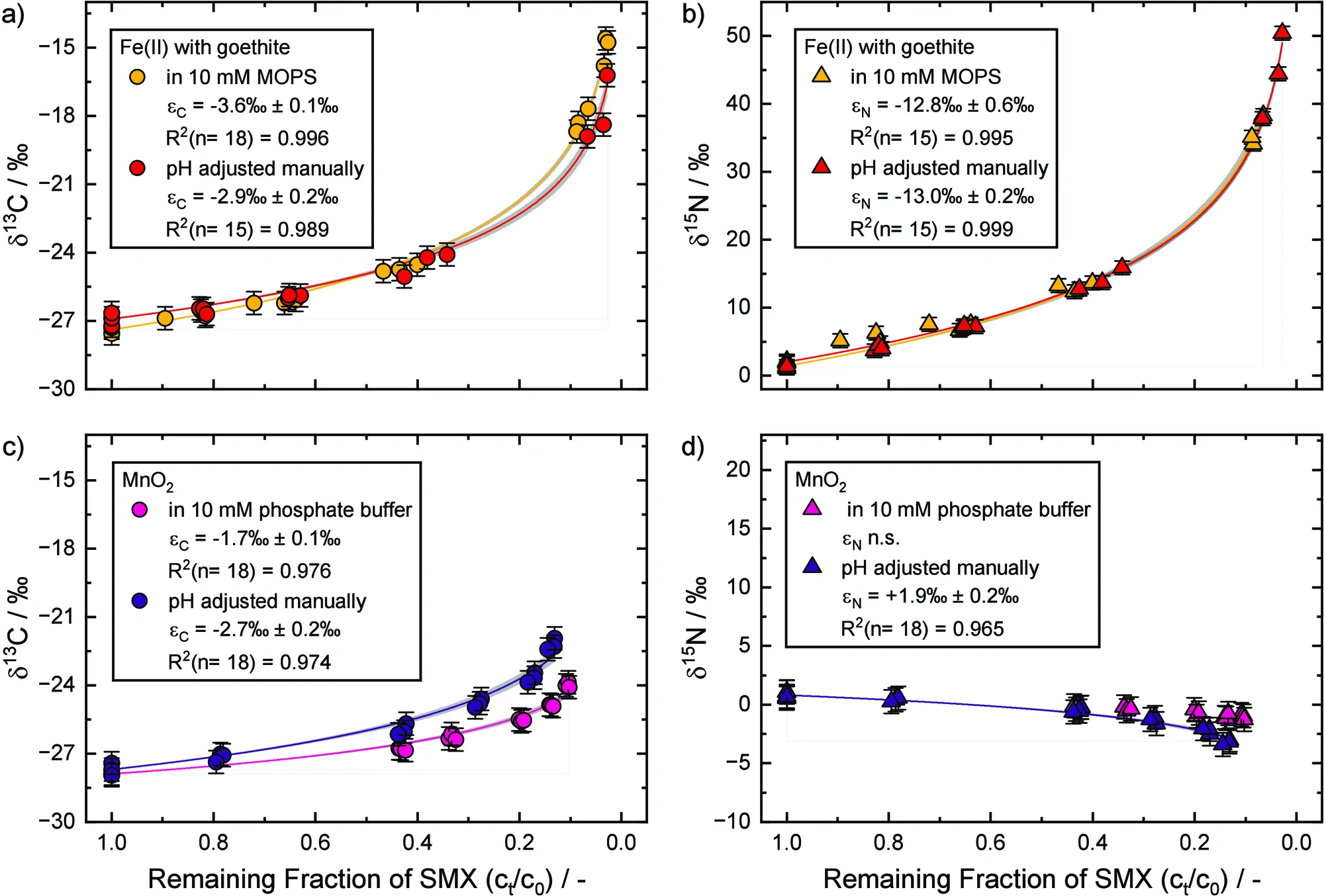
(a) Carbon and (b) nitrogen isotope fractionation of SMX in Fe­(II)-amended goethite suspensions in the presence and absence of MOPS buffer at pH 7.0 ± 0.2; (c) carbon and (d) nitrogen isotope fractionation of SMX in MnO_2_ suspensions in the presence and absence of phosphate buffer at pH 3.0 ± 0.2; noting that the first data points for each experimental triplicate correspond to *c*_t_/*c*_0_ = 1 and are obscured by overlapping data points, e.g., pink symbols in panel d at *c*_t_/*c*_0_ = 1 and 0.4; error bars correspond to 95% CIs associated with the respective isotope analysis (±0.5‰ for δ^13^C analysis and ±1‰ for δ^15^N analysis, noting that in (b) the error bars are smaller than the markers). Lines in the respective color represent the regressions according to eq 3, together with their 95% CIs in gray. The respective ε and associated uncertainties were obtained by linearizing eq 3 (Figure S4). n.s. = not significant.

### Reductive Transformation of SMX with Fe­(II) and Goethite

#### Linking Isotope Fractionation to the Transformation Pathway

During reductive SMX transformation, four TPs formed simultaneously, including three tentatively identified TPs (TP271, TP255, and TP253) to level 3 of Schymanski et al.^51^ and sulfanilamide, which was confirmed by using an authentic standard (Tables S3 and S4). TP255 and TP253 showed significantly larger peak area ratios than TP271 and sulfanilamide (Figure 2 and Figures S5 and S6b). TP255 may arise from ring opening of the isoxazole moiety (pathway A, Scheme 1), whereas TP253 would result from a rearrangement of the isoxazole moiety via an azirine ring (pathway B; Scheme 1). TP271 formation would initially follow the same pathway as TP253, but the azirine would be hydrolytically cleaved. These TPs have been previously found during anoxic SMX transformation.^13,15,28^ The formation pathway to the detected sulfanilamide remained unclear (pathway C, Scheme 1); however, the formation of all four TPs was proposed to be initiated by a single-electron transfer that cleaved the N–O bond in the isoxazole moiety, leading to a common intermediate (Scheme 1).

**Scheme 1:**
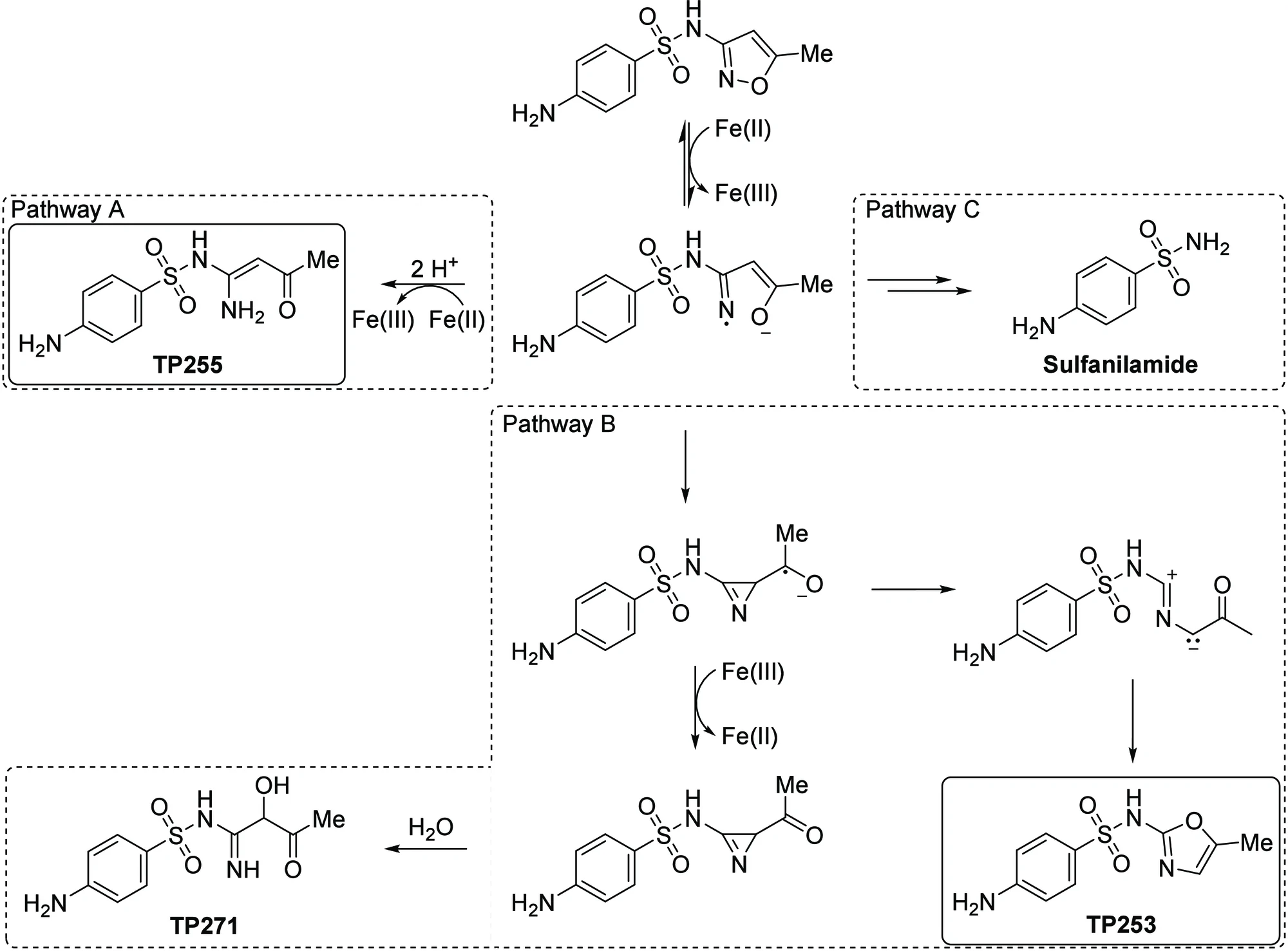
Proposed Mechanism of SMX Transformation with Fe­(II)-Amended Goethite, Highlighting the Two Major Transformation Products

**Figure 2:**
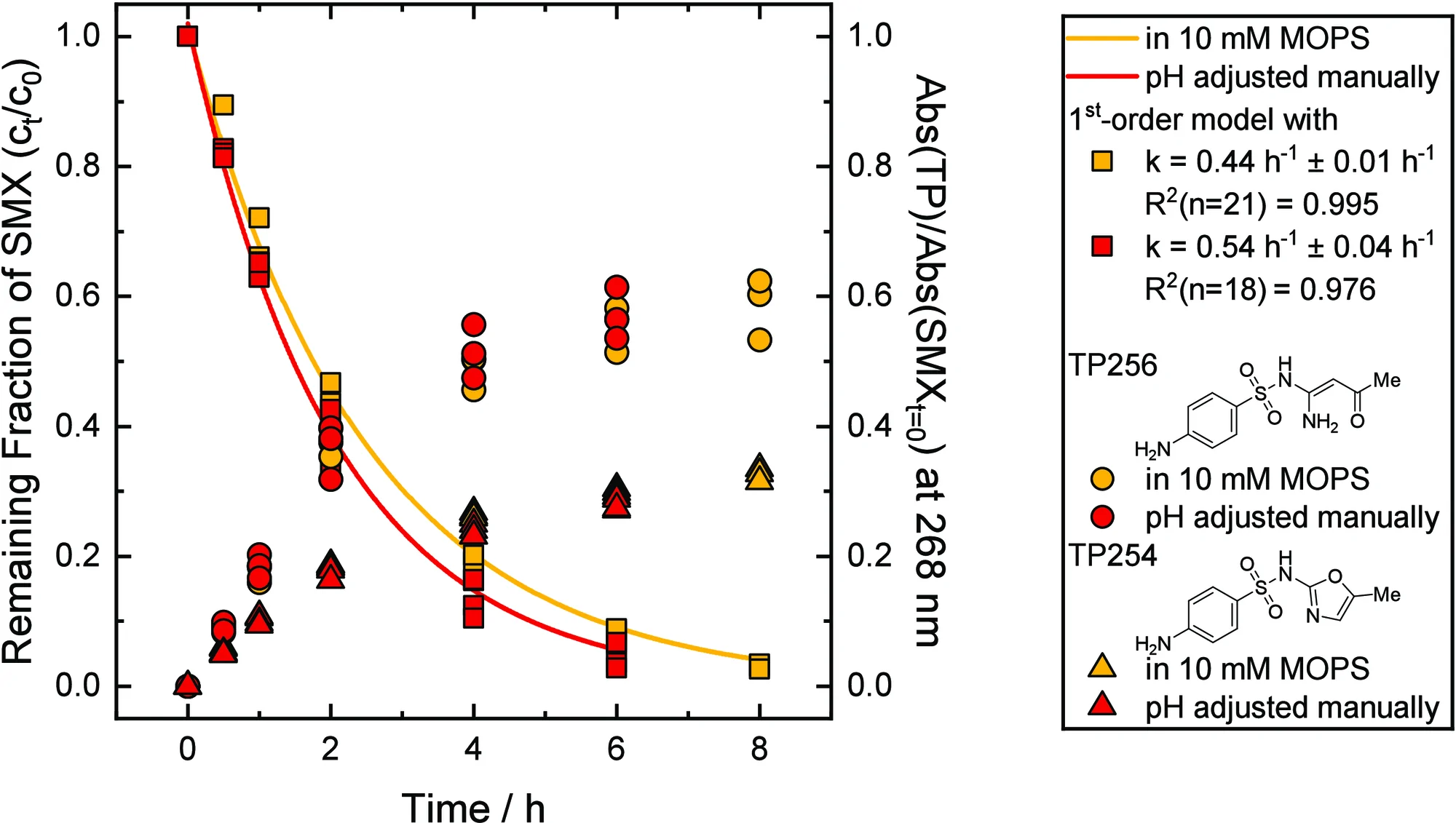
Time course for the abiotic transformation of SMX in Fe­(II)-amended aqueous goethite suspensions in a 10 mM MOPS buffer and in the absence of a MOPS buffer, with manual pH adjustment throughout the experiments, both at pH 7.0 ± 0.2. The first-order rate constants *k* were determined by linearizing eq 5 (Figure S6a). The formation of the two major TPs, TP255 and TP253, throughout the experiment with and without a buffer at pH 7.0 ± 0.2 is represented as relative peak area ratios (peak area of TP at a certain time point over the initial SMX peak area) at the wavelength of 268 nm.

Hence, during reductive transformation, the observed normal nitrogen isotope fractionation (ε_N_ = −12.8‰ ± 0.6‰ and ε_N_ = −13.0‰ ± 0.2‰, in the presence and absence of buffer, respectively; Figure 1b), likely arises from the initial reaction step, the N–O bond cleavage forming the SMX radical anion. A contribution may also arise from the subsequent paths after this reaction step, since the formation of both major TPs involves nitrogen atoms: TP255 involves the protonation of an iminyl radical, and TP253 involves a C–N bond formation resulting in the azirine intermediate. In parallel, the reductive transformation of SMX showed pronounced carbon isotope fractionation in the presence and absence of MOPS buffer. This is unexpected at first, since the initial reaction step, the formation of the SMX radical anion, does not involve any carbon bond cleavage or formation. Therefore, we hypothesize that the common initial reaction step is likely reversible so that the observed carbon isotope effects can arise from subsequent steps that do involve bond changes at carbon atoms (i.e., pathway B, Scheme 1). We note that the same two major TPs, TP253 and TP255, have also been detected during the anaerobic transformation of SMX by *Desulfovibrio vulgaris* Hildenborough, which was accompanied by an even greater normal carbon isotope effect (ε_C_ = −5.8‰ ± 0.7‰) than found in our study.^28^ This agreement indicates that both the product formation and the trend in carbon isotope fractionation observed in our study are likely representative of reductive SMX transformation. Because of the complexity of the scenarios and the multiple molecular positions involved (see discussion below), we did not attempt to calculate position-specific apparent kinetic isotope effects (AKIEs). The same consideration guided us in the MnO_2_-mediated oxidative transformation further below.

#### Subtle Effects from the Presence of a Buffer

For the reductive transformation of SMX, different first-order reaction rate constants in the presence and the absence of the MOPS buffer (*k* = 0.44 h^–1^ ± 0.01 h^–1^ and *k* = 0.54 h^–1^ ± 0.04 h^–1^, respectively; Figure 2) were observed, which were both of the same order of magnitude as previously reported for this transformation process.^15^ Consistent with our findings, a faster reaction in the absence of a buffer has previously been observed during the reductive transformation of tetrachloromethane and has been explained by the release of Fe­(II) species from the mineral surface, induced by the buffer.^47^ A prominent influence of pH fluctuations on SMX speciation and, consequently, on reaction kinetics caused by manual pH adjustment in experiments without a buffer is unlikely, because the observed pH fluctuations only caused subtle changes in the amount of SMX in its neutral form (considering a p*K*_a_ value of 5.7,^14^ at pH 7, approximately 5% of SMX exists in its neutral form, whereas at pH 6.8, this increases to about 7%). Further, a previous study showed an opposite effect, that, under these reaction conditions, SMX transformation rate constants increased rather than decreased with increasing pH.^15^ A direct comparison of the experiments with and without MOPS buffer also revealed a subtle shift in the TP distribution. While TP255 formation did not change between the two systems, in the presence of the MOPS buffer, 1.2 times more TP253 was observed than in its absence (Figure S6b). Additionally, the minor TP271 was formed to a greater extent in the presence of the MOPS buffer, indicating a greater formation of the TPs via pathway B (Figure S5a).

Finally, a subtle effect was also observed for carbon isotope fractionation in the presence and absence of MOPS buffer (ε_C_ = −3.6‰ ± 0.1‰ and ε_C_ = −2.9‰ ± 0.2‰, respectively; Figure 1a). One possible explanation is a shift in product distribution, which would lead to a different weight of the isotope effects associated with the parallel pathways starting from the common intermediate. However, the changes in TP formation and, consequently, the respective pathways were subtle and most probably cannot explain the observed differences. A second possible explanation is that the initial first step, which does not involve a carbon-bond cleavage, was more strongly rate-determining in the absence of a buffer, leading to a stronger representation of the first step’s negligible carbon isotope effect in the observable fractionation. However, this phenomenon - blockage of surface sites that may slow electron transfer – may rather be expected in the presence than the absence of a buffer. A third possible explanation would, finally, be a change in transition state structures caused by the presence of the MOPS buffer, which could alter the surface chemistry of goethite and thereby change the energy differences between isotopologues during the initial formation of the SMX radical anion.^53^

### Oxidative Transformation of SMX with MnO_2_

#### Linking Isotope Fractionation to the Transformation Pathway

During the transformation reaction with MnO_2_, traces of tentatively identified oxidation products were observed, including N–H oxidation (TP283), self-coupling (TP504), formation of a diazo compound (TP295), and oxidation of the aromatic ring of SMX (TP218, TP220, TP260, TP281, and TP325) (Tables S5 and S6), confirming that the transformation of SMX with MnO_2_ was indeed oxidative. The tentatively identified structures for the TPs showed a breakdown and oxidation of the aromatic ring (TP218, TP220, TP260, TP281, and TP325), which were confirmed by experiments with ^13^C_10_-labeled SMX. Evaluating the masses of labeled TPs in comparison to the same TPs in an experiment with SMX at natural abundance made it possible to uniquely determine the number of carbon atoms in each TP. Since TPs detected by high-resolution mass spectrometry were present only at trace levels, we performed additional analyses to close the mass balance. First, we alternatively stopped the MnO_2_ transformation reaction by either filtration or dissolution of the MnO_2_ particles via the addition of oxalic acid to exclude sorption of TPs to the MnO_2_ surface and detected the same TPs in both samples. Second, we analyzed nonpurgeable dissolved organic carbon of filtered samples after 8 days, when most SMX had been transformed. A decrease of only 14% from the initial amount indicated that most of the TPs remained in solution but were undetectable by the LC-MS employed. Putative structures could be low-molecular-weight TPs arising from the breakdown of the aromatic ring (TP218, TP220, TP260, TP281, and TP325). The decrease in nonpurgeable organic carbon indicated the formation of small amounts of CO_2_, which was confirmed by qualitatively tracing the incorporation of ^13^C from ^13^C_10_-labeled SMX into CO_2_ in a closed reaction system (Figure S7). Finally, during the reaction, a white precipitate formed and floated on the surface of the reaction suspension. This could arise from the formation of an SMX polymer.^54^ All of these lines of evidence confirm that, indeed, oxidative transformation of SMX was at work.

This transformation of SMX with MnO_2_ showed inverse nitrogen isotope fractionation (ε_N_ = +1.9‰ ± 0.2‰ in the absence of buffer; Figure 1d), in a similar way as for the transformation of substituted anilines with MnO_2_ and SMX with radicals, whereby the initial reaction step was proposed to occur at the aniline, forming an aniline radical cation.^30,33^ The observed inverse nitrogen isotope fractionation supports the hypothesis of an oxidative transformation initiated by the one-electron oxidation of SMX to MnO_2_, forming a SMX radical cation delocalized over the aromatic ring, as also indicated by the traces of TPs. During MnO_2_ oxidation of substituted anilines, inverse nitrogen isotope fractionation was previously observed only at pH values that preclude any contribution from ^15^N-protonation isotope effects. In our study, the experiments were likewise conducted at pH conditions that avoid contributions of ^15^N-protonation equilibria to the observed kinetic isotope effect.^33^ We note that SMX contains three nitrogen atoms in total, so the two nitrogen atoms outside the aniline ring could potentially dilute the inverse isotope effect or even cause an opposite effect to the observed isotope fractionation. In the absence of position-specific isotope information, however, these effects cannot be resolved. For carbon, a normal isotope effect was observed (ε_C_ = −1.7‰ ± 0.1‰ and ε_C_ = −2.7‰ ± 0.2‰, in the presence and absence of buffer, respectively; Figure 1c). As recently reported, the reaction of SMX with persulfate radicals at the same pH value also exhibited inverse nitrogen isotope fractionation and normal carbon isotope fractionation; however, the magnitudes of these effects were significantly lower than those observed in this study for MnO_2_ oxidation.^30^ Overall, analogous to aniline transformation with MnO_2_,^33^ we postulate that the reaction mechanism is initiated by the formation of a radical intermediate upon one-electron oxidation, which is delocalized over the aromatic ring.

#### Biphasic Reaction Kinetics

The transformation of SMX with MnO_2_ reached a conversion of 90% (remaining fraction of 10%) after 42 days in the presence of a phosphate buffer and after 28 days in the absence of the buffer (Figure S8). In both experiments, similar trends in concentration over time were observed, with a rapid initial transformation followed by a decreasing reaction rate (Figure S8b). Similar to the reductive transformation, the buffer affected the reaction kinetics. It has been hypothesized that an initial, faster reaction rate constant in the presence of a phosphate buffer could be due to stabilization of reactive manganese species by the buffer, as brought forward for the oxidation of bisphenol A by permanganate.^55^ Biphasic disappearance kinetics of organic compounds with MnO_2_ have also been found for substituted anilines^32,33^ and diclofenac.^56^ However, in the case of anilines, the biphasic reaction kinetics were attributed to a decrease in MnO_2_ reactivity over time as the mineral surface became progressively reduced rather than to changes in the underlying aniline reaction mechanism.^33^ Hence, we did not consider the possibility of two individual mechanisms in the interpretation of the isotope fractionation.

### Dual-Element Isotope Analysis of Oxidative and Reductive Transformation of Sulfamethoxazole

In this study, we found that dual-element isotope slopes of carbon and nitrogen vary significantly between oxidative transformation by MnO_2_ (Λ_N/C_ = −0.7 ± 0.2, Figure 3), and reductive transformation by Fe­(II)-amended goethite suspensions (Λ_N/C_ = 3.6 ± 0.2 and 4.6 ± 0.2, Figure 3), showing that these two pathways can be differentiated using a dual-element isotope approach. When comparing the dual-element isotope slopes to those reported for oxidation with heat-activated persulfate at pH 3 and pH 7 (Λ_N/C_ = −0.5 ± 0.2 and Λ_N/C_ = −0.4 ± 0.2),^30^ the trends are not significantly different, indicating that similar reaction steps are present, which, however, is not the case for the reaction with heat-activated persulfate at pH 10 (Λ_N/C_ = +0.2 ± 0.1).^30^ In contrast, photolysis (Λ_N/C_ = −1.3 ± 0.2 (pH 3) and −1.5 ± 0.2 (pH 7))^29^ can be distinguished from the reductive and oxidative processes by dual-element isotope analysis. To gain further insight into specific reaction sites and associated isotope effects, recent advances in position- or fragment-specific isotope analysis by Orbitrap mass spectrometry delineate a promising way ahead.^48,57^

**Figure 3:**
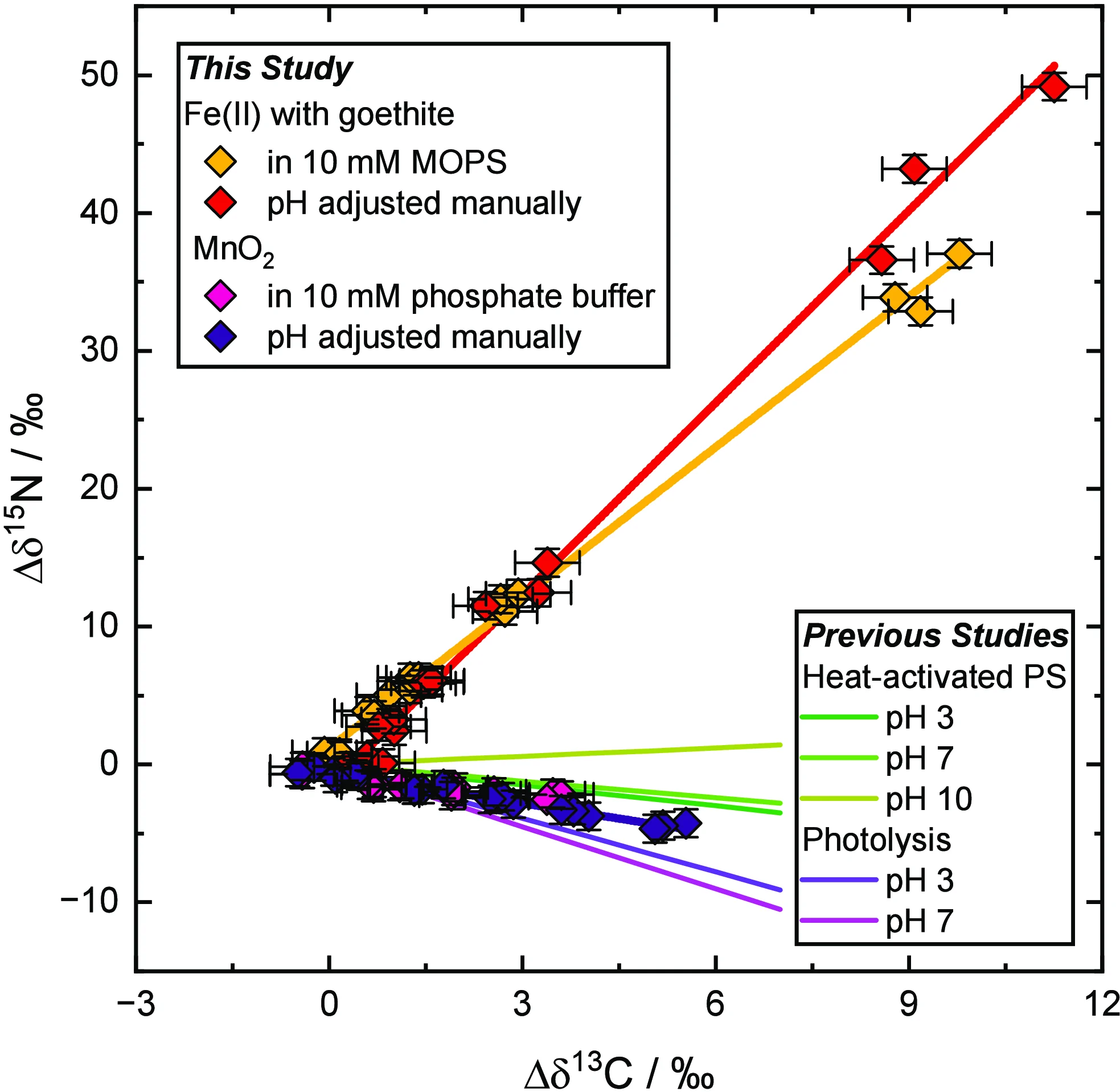
Dual-element isotope plot for the two abiotic transformation processes of SMX obtained in this study with dual-element isotope slopes on transformation for previous studies with heat-activated persulfate (PS) (green)^30^ and photolysis (purple)^29^ at different pH. Error bars correspond to typical uncertainties associated with the respective isotope analysis (±0.5‰ for δ^13^C analysis and ±1‰ for δ^15^N analysis). Lines in the respective color represent the York regressions.

### Environmental Significance

To extrapolate these results from the laboratory to the field, potential confounding effects such as speciation and sorption to mineral surfaces or organic matter may need to be considered. With p*K*_a_ values of approximately 1.6 and 5.7,^14^ SMX occurs primarily in its neutral form under mildly acidic conditions (as in the MnO_2_ experiments) and predominantly as an anion at neutral to alkaline pH (as in the Fe­(II)/goethite experiments). This is typical of many soils, groundwater systems, surface waters, and wastewater treatment processes. In its neutral form, SMX exhibits a relatively low organic carbon–water partition coefficient (log *K*oc ≈ 0.48–0.62),^58^ indicating substantial mobility in soils and sediments. Under neutral and alkaline conditions, where SMX is predominantly anionic, sorption onto negatively charged mineral and organic surfaces is even expected to be further reduced, thereby enhancing its transport through aquatic and subsurface environments. Consequently, SMX is expected to be little affected by sorption, and to encounter diverse redox-active minerals, creating opportunities for both oxidative and reductive abiotic transformation as investigated in this study. Consequently, our study shows the potential of isotope fractionation to estimate the extent of contaminant transformation in the environment and during engineered treatment processes. This is particularly valuable when parent compound concentration measurements or TP detection are inconclusive, as was the case with the transformation of SMX by MnO_2_ in our batch experiments. This offers the perspective of distinguishing between reductive and oxidative transformation in subsurface environments where MnO_2_ and iron minerals can naturally occur, and SMX transformation may potentially be driven under different redox conditions. Another promising application is the identification of the mechanisms behind SMX elimination in wastewater treatment plants, where reductive and oxidative treatment may occur sequentially. For such practical applications, CSIA on micropollutants is currently challenged by low environmental concentrations (sub-μg L^–1^), since CSIA requires significant amounts of purified samples. Advances are expected from ongoing efforts in sample preparation and analytical instrumentation, including optimized GC-IRMS systems,^59,60^ preparative HPLC cleanup procedures,^61^ novel sorbent materials that reduce coenrichment of the matrix interferences,^62^ and passive samplers.^63–65^

## Supplementary Material



## Notes

Chemicals and solvents used, synthesis procedure and NMR characterization of each reaction step of the synthesis of ^13^C_10_ sulfamethoxazole, characterization of the minerals used, control experiments for the transformation reactions of sulfamethoxazole, GC-IRMS measurements, details on the LC-ESI-Orbitrap method, Rayleigh plots, transformation products formed during the reductive transformation of sulfamethoxazole by Fe­(II)-amended goethite suspensions, reaction kinetics of the transformation of sulfamethoxazole by Fe­(II)-amended goethite suspensions, transformation products formed during the oxidative transformation of sulfamethoxazole by MnO_2_ suspensions, GC-IRMS measurement for qualitative detection of ^13^CO_2_ during the oxidative transformation of sulfamethoxazole, transformation kinetics of sulfamethoxazole by MnO_2_ suspensions (PDF)
