# Supplementary material for: Insight from Carbon and Nitrogen Isotope Fractionation of Sulfamethoxazole during Oxidative vs Reductive Transformation
Source: Environ Sci Technol. 2026 Oct 1;60(40):29066–76. doi: 10.1021/acs.est.6c07718 (PMC13674070; doi:10.1021/acs.est.6c07718)
Supplement: Supplementary file 1 [file es6c07718_si_001.pdf]

Supporting Information for *Environmental Science and Technology*

Insight from Carbon and Nitrogen Isotope Fractionation of Sulfamethoxazole during Oxidative vs Reductive Transformation

Aoife Canavan[a,#], Christopher Dirr[a,#], Martin Elsner[a,*]

[a]Technical University of Munich, TUM School of Natural Sciences, Department of Chemistry, Chair of Analytical Chemistry and Water Chemistry, Lichtenbergstraße 4, 85748 Garching, Germany

[#]shared first authorship: these authors contributed equally to the manuscript

*Corresponding author: Martin Elsner
Address: Lichtenbergstraße 4, 85748 Garching, Germany
Phone: +49 (89) 289 - 54500
E-mail: m.elsner@tum.de

Contents

S1 Chemicals and Solvents ... 2
S2 Synthesis Procedure and NMR Characterization of Each Reaction Step of the Synthesis of $^{13}C_{10}$ Sulfamethoxazole ... 3
S3 Characterization of the Minerals Used ... 9
S4 Control Experiments for the Transformation Reactions of Sulfamethoxazole ... 10
S5 GC-IRMS Measurements ... 10
S6 Details on the LC-ESI-Orbitrap Method ... 11
S7 Rayleigh-Plots ... 12
S8 Transformation Products Formed during the Reductive Transformation of Sulfamethoxazole by Fe(II)-Amended Goethite Suspensions ... 13
S9 Reaction Kinetics of the Transformation of Sulfamethoxazole by Fe(II)-Amended Goethite Suspensions ... 14
S10 Transformation Products Formed during the Oxidative Transformation of Sulfamethoxazole by MnO_2 Suspensions ... 15
S11 GC-IRMS Measurement for Qualitative Detection of $^{13}CO_2$ during the Oxidative Transformation of Sulfamethoxazole ... 19
S12 Transformation Kinetics of Sulfamethoxazole by MnO_2 Suspensions ... 19
S13 References ... 19

S1 Chemicals and Solvents

The ultrapure water (18.2 MΩ cm at 25 °C) used was from a Milli-Q Reference water purification system (Merck Millipore, USA). Solutions of hydrochloric acid and sodium hydroxide (1 M) were prepared by mixing or dissolving the respective amounts of the concentrated acid or pellets in ultrapure water. For LC-ESI-Orbitrap measurements, LC-MS methanol was used; otherwise, methanol for HPLC was used.

Table S1: Detailed description of analytical standards, chemicals, and solvents used.

	Additional Information	Supplier
Analytical Standards		
3-Amino-5-methylisoxazole	≥97%,	Sigma-Aldrich
IAEA-600	Caffeine	
Sulfamethoxazole (SMX)	BCCH2594	Sigma-Aldrich
Sulfanilamide	Analytical standard	Vetranal
USGS62	Caffeine	
USGS63	Caffeine	
Chemicals		
Bayferrox 910 (Goethite)	α-FeOOH	Lanxess
Formic acid	≥ 99.0%, LC-MS grade	Carl Roth
Hydrochloric acid	puriss. p.a., ACS reagent, ≥37%	Sigma-Aldrich
Iron powder	nanopowder, 40-60 nm particle size, 99% trace metals basis	Sigma-Aldrich
Manganese(II)-chloride tetrahydrate	GR for analysis	Sigma-Aldrich
Monosodium phosphate	≥99.0%	FLUKA
3-(N-Morpholino)propanesulfonic acid	≥99.5%	Sigma-Aldrich
Oxalic acid	≥99%	Sigma-Aldrich
Phosphoric acid	≥85%, for analysis	Sigma-Aldrich
Sodium chloride	ACS reagent, ≥99%	Sigma-Aldrich
Sodium hydroxide	≥98%, pellets, anhydrous	Sigma-Aldrich
Sodium permanganate	40wt.% in water	Sigma-Aldrich
Trimethylsilyl diazomethane	1.8 M–2.4 M in hexanes	Thermo Fisher Scientific
Solvents		
Acetonitrile	for HPLC, ≥99.9%	Sigma-Aldrich
Ethyl acetate	for HPLC, ≥99.9%	Sigma-Aldrich
Methanol	for HPLC, ≥99.9%	Sigma-Aldrich
Methanol	hypergrade for LC-MS	Sigma-Aldrich
Labeled Starting Material for $^{13}C_{10}$-SMX according to S2		
Acetanilid-(ring-$^{13}C_6$)	EW1074, 99 atom% ^{13}C	Sigma-Aldrich
Acetonitrile-$^{13}C_2$	MBBD4066, 99 atom% ^{13}C, contains copper as a stabilizer	Sigma-Aldrich
Ethyl acetate-1,2-$^{13}C_2$	MBBD7143, 99 atom% ^{13}C	Sigma-Aldrich
Ethyl acetate-1,2-$^{13}C_2$	MBBD3467, 99 atom% ^{13}C	Sigma-Aldrich

S2 Synthesis Procedure and NMR Characterization of Each Reaction Step of the Synthesis of $^{13}C_{10}$ Sulfamethoxazole

The synthesis of $^{13}C_{10}$-sulfamethoxazole was performed according to Canavan et al.[1], using starting materials with ^{13}C labels at positions retained in the final SMX. The yields for each reaction step are provided below, and the respective intermediates and final product were characterized by ^{1}H and ^{13}C-NMR.

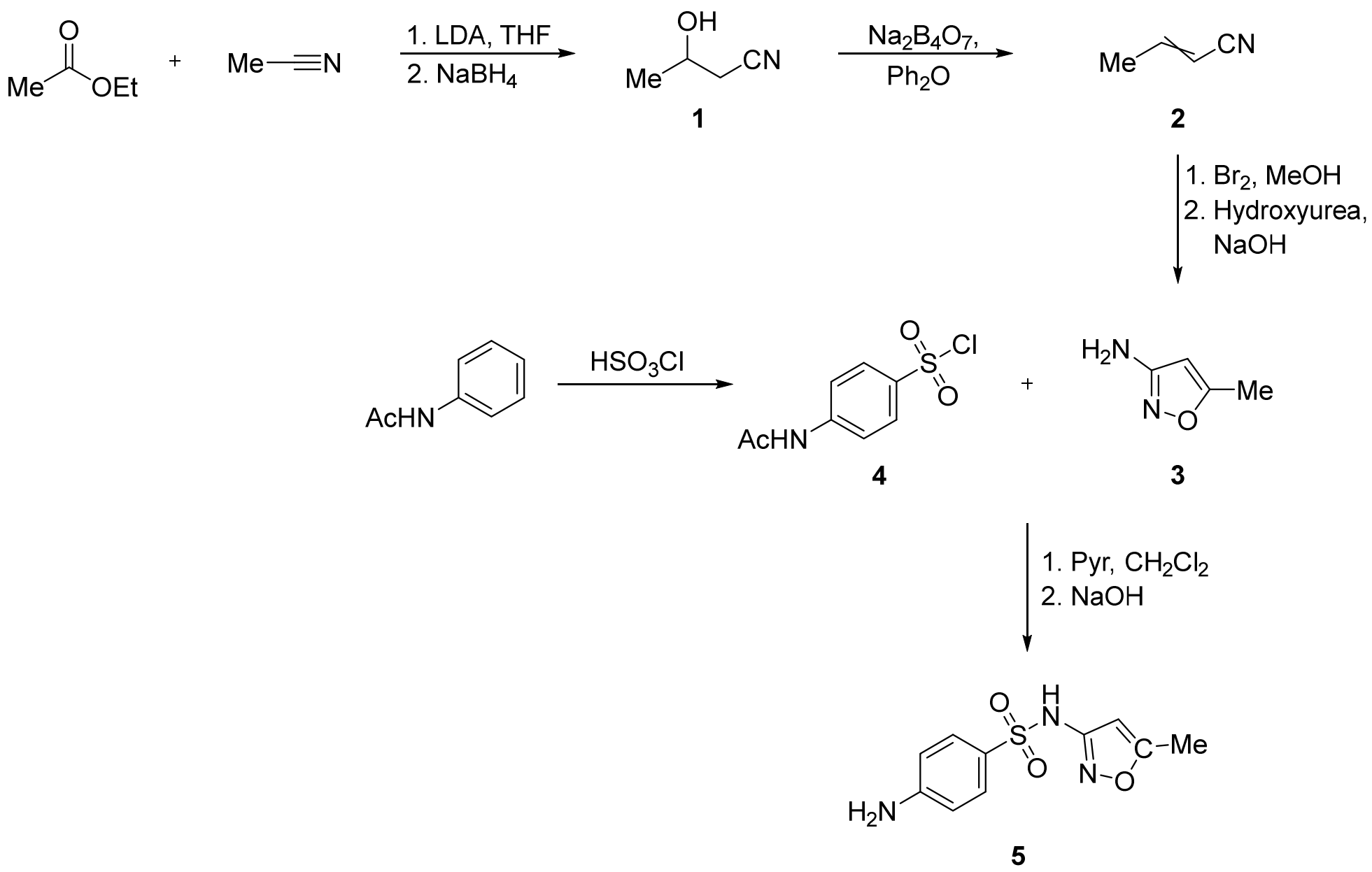

Scheme S1: Overview of the performed reactions for the synthesis of $^{13}C_{10}$-Sulfamethoxazole.

3-Hyroxybutyronitrile-1,2,3,4-$^{13}C_4$ (**1**) (3.30 g, 37.0 mmol, 73%):

^{1}H-NMR (500 MHz, $CDCl_3$): δ (ppm) = 1.22-1.50 (3 H, m, CH_3), 2.03 (1 H, s br, OH), 2.33-2.71 (2 H, m, CH_2), 3.99-4.34 (1 H, m, CH).

^{13}C-NMR (101 MHz, $CDCl_3$): δ (ppm) = 22.9 (dd, J = 38.7, 3.1 Hz, CH_3), 27.6 (ddd, J = 56.9, 36.8, 1.5 Hz CH_2), 64.3 (ddt, J = 38.3, 36.4, 1.9 Hz, CH), 117.6 (dq, J = 57.0, 2.9 Hz, CN).

Crotonitrile-1,2,3,4-^{13}C (**2**) (56%, 601 mg, 8.46 mmol):

Z-isomer:

^{1}H-NMR (500 MHz, $CDCl_3$): δ (ppm) = 2.05 (3 H, dqt, $^{1}J_{C\text{-}H}$ = 128.3 Hz, $^{3}J_{H\text{-}H}$ = 7.2 Hz, $^{4}J_{H\text{-}H}$ = 1.7 Hz, CH_3), 5.14-5.55 (1 H, m, C*H*-CH_3), 6.36-6.74 (1 H, m, C*H*-CN).

^{13}C-NMR (101 MHz, $CDCl_3$): δ (ppm) = 17.6 (ddd, J = 41.6, 4.6, 2.0 Hz, CH_3), 100.0-101.2 (m, *C*H-CN), 116.1 (dd, J = 77.6, 4.8 Hz, CN), 149.0-150.7 (m, *C*H-CH_3).

E-isomer:

^{1}H-NMR (500 MHz, $CDCl_3$): δ (ppm) = 1.92 (3 H, dqt, $^{1}J_{C\text{-}H}$ = 129.0 Hz, $^{3}J_{H\text{-}H}$ = 7.2 Hz, $^{4}J_{H\text{-}H}$ = 1.7 Hz, CH_3), 5.14-5.55 (1 H, m, C*H*-CH_3), 6.52-6.93 (1 H, m, C*H*-CN).

^{13}C-NMR (101 MHz, $CDCl_3$): δ (ppm) = 19.1 (ddd, *J* = 41.6, 8.2, 2.2 Hz, CH_3), 100.8-102.0 (m, *C*H-CN), 117.6 (dd, *J* = 79.5, 8.5 Hz, CN), 150.7-151.8 (m, *C*H-CH_3).

3-Amino-5-methyl-isoxazole-3,4,5,6-$^{13}C_4$ (**3**) (70%, 2.15 g, 21.1 mmol):

^{1}H-NMR (500 MHz, DMSO-d_6): δ (ppm) = 2.05-2.33 (3 H, m, CH_3), 5.39 (2 H, s br, NH_2), 5.37-5.75 (1 H, m, CH).

^{13}C-NMR (101 MHz, DMSO-d_6): δ (ppm) = 11.9 (ddd, *J* = 51.0, 6.7, 3.3 Hz, CH_3), 94.3 (ddd, *J* = 72.2, 56.0, 6.6 Hz, CH), 164.0 (dt, *J* = 56.0, 3.1 Hz, C-NH_2), 167.3 (ddd, *J* = 72.4, 51.1, 3.1 Hz, C-O-N).

N-Acetylsulfanilyl chloride-1,2,3,4,5,6-$^{13}C_6$ (**4**) (70%, 7.14 g, 29.8 mmol):

^{1}H-NMR (500 MHz, DMSO-d_6): δ (ppm) = 2.03 (3 H, s, CH_3), 7.34 (2 H, m, CH_{ar}), 7.63 (2 H, m, CH_{ar}), 10.0 (1 H, s br, NH).

Sulfamethoxazole-1,2,3,4,5,6,7,8,9,10-$^{13}C_{10}$ (**5**) (60%, 1.57 g, 5.97 mmol):

^{1}H-NMR (500 MHz, DMSO-d_6): δ (ppm) = 2.28 (3 H, ddd, $^{1}J_{C\text{-}H}$ = 130.0 Hz, $^{2}J_{C\text{-}H}$ = 7.1 Hz, $^{3}J_{C\text{-}H}$ = 3.1 Hz, CH_3), 6.08 (1 H, t, $^{2}J_{C\text{-}H}$ = 5.6 Hz, NH_2), 6.08 (1 H, ddd, $^{1}J_{C\text{-}H}$ = 184.8 Hz, $^{2}J_{C\text{-}H}$ = 9.7 Hz, $^{3}J_{C\text{-}H}$ = 3.6 Hz, CH), 6.58 (2 H, dm, $^{1}J_{C\text{-}H}$ = 166.4 Hz, CH_{ar}), 7.46 (2 H, dm, $^{1}J_{C\text{-}H}$ = 161.9 Hz, CH_{ar}), 10.91 (1 H, s br, NH).

^{13}C-NMR (101 MHz, DMSO-d_6): δ (ppm) = 12 (ddd, *J* = 50.7, 6.5, 3.0 Hz, CH_3), 95 (ddd, *J* = 70.7, 59.8, 6.5 Hz, CH), 112 (*J* = 59.2, 6.0 Hz, C_{ar}), 125 (m, C_{ar}), 128 (m, C_{ar}), 153 (td, *J* = 59.3, 8.4 Hz, C_{ar}), 158 (dt, *J* = 59.6, 3.5 Hz, *C*-CH_3), 170 (ddd, *J* = 70.7, 50.7, 3.7 Hz, *C*-NH).

3-Hyroxybutyronitrile-1,2,3,4-^{13}C (1)

^{1}H-NMR (500 MHz, $CDCl_3$):

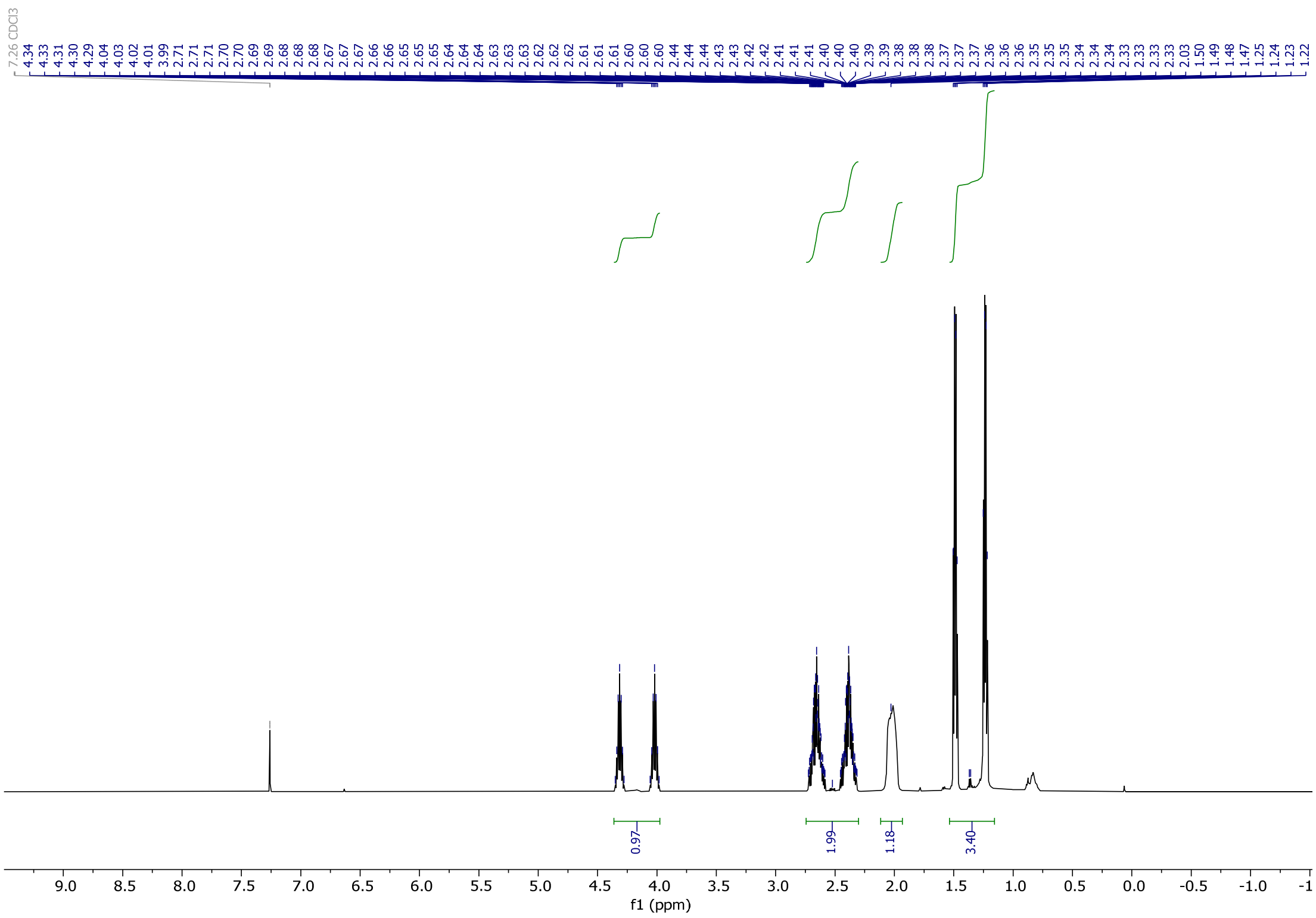

^{13}C-NMR (101 MHz, CDCl$_3$):

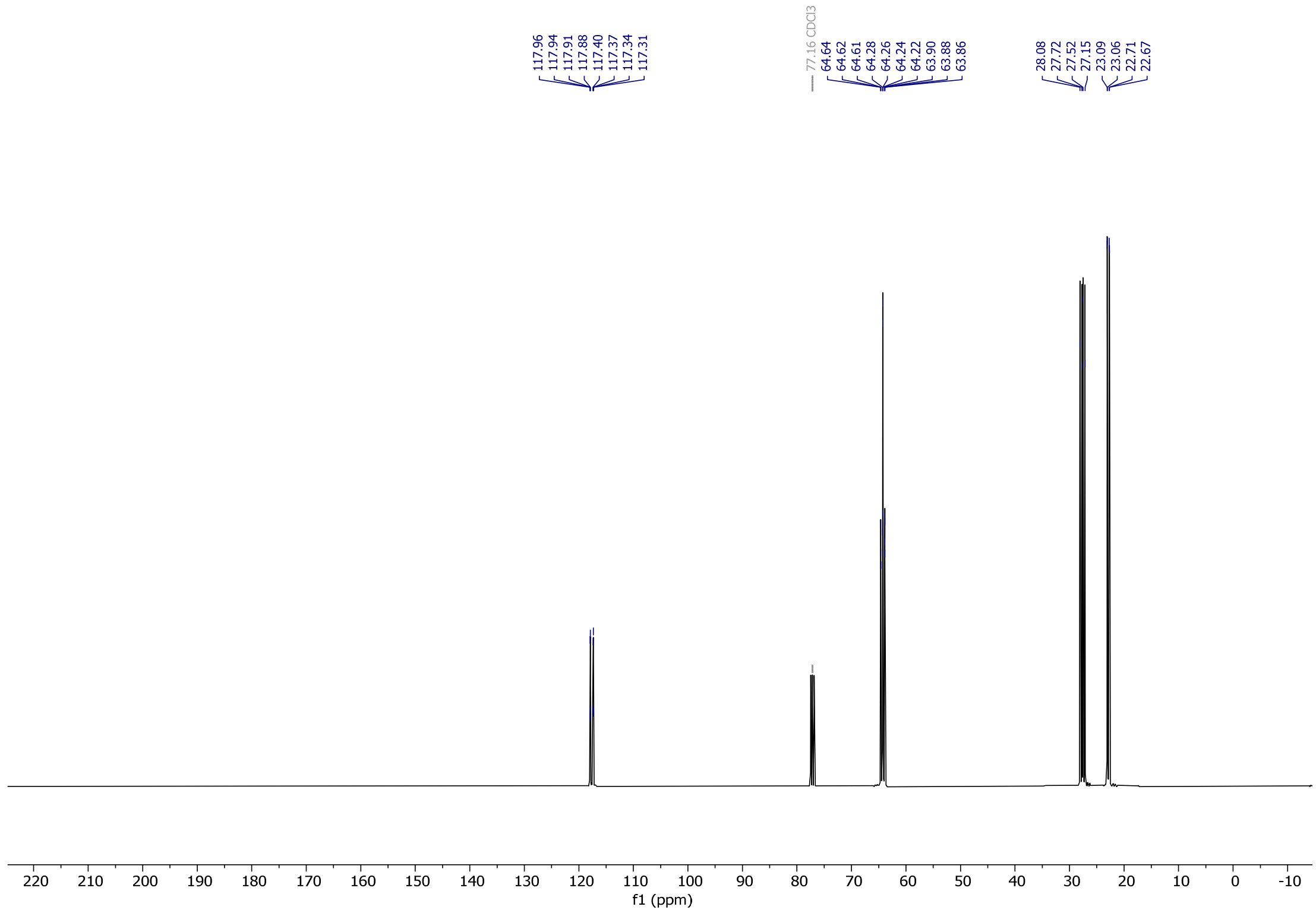

Crotononitrile-1,2,3,4-^{13}C (2)

^{1}H-NMR (500 MHz, CDCl$_3$):

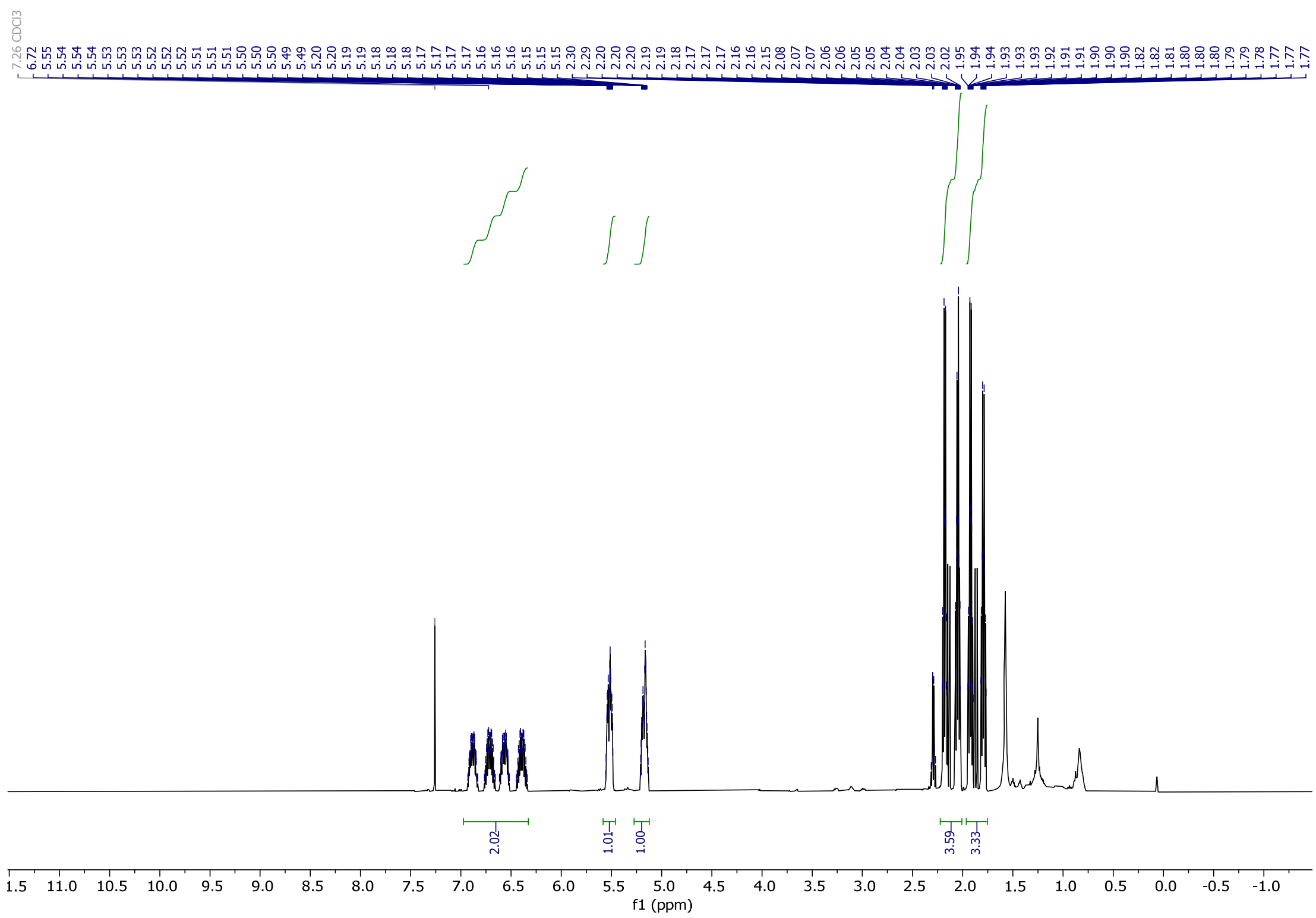

^{13}C-NMR (101 MHz, $CDCl_3$):

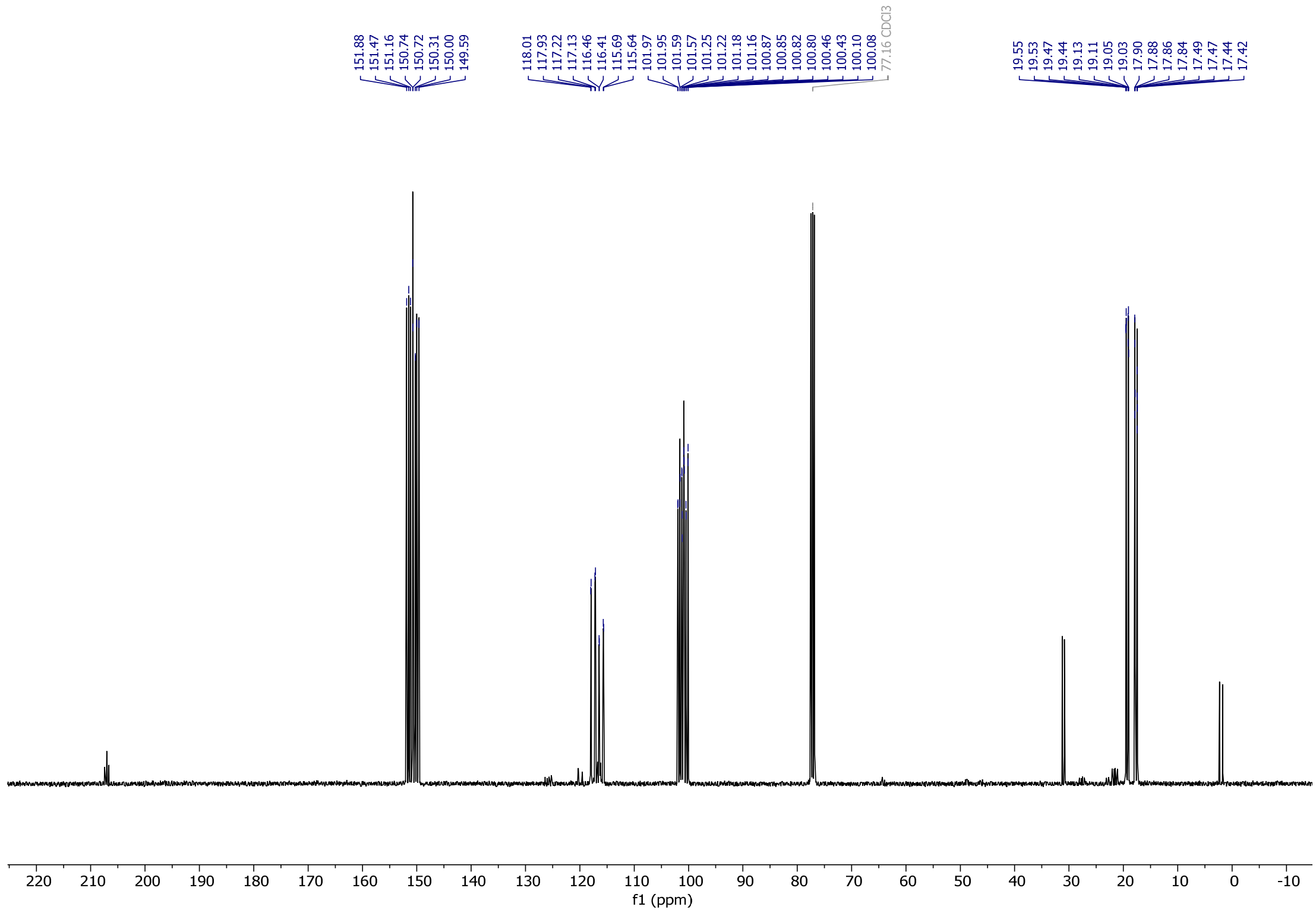

3-Amino-5-methyl-isoxazole-3,4,5,6-$^{13}C_4$ (3)

^{1}H-NMR (500 MHz, DMSO-d_6):

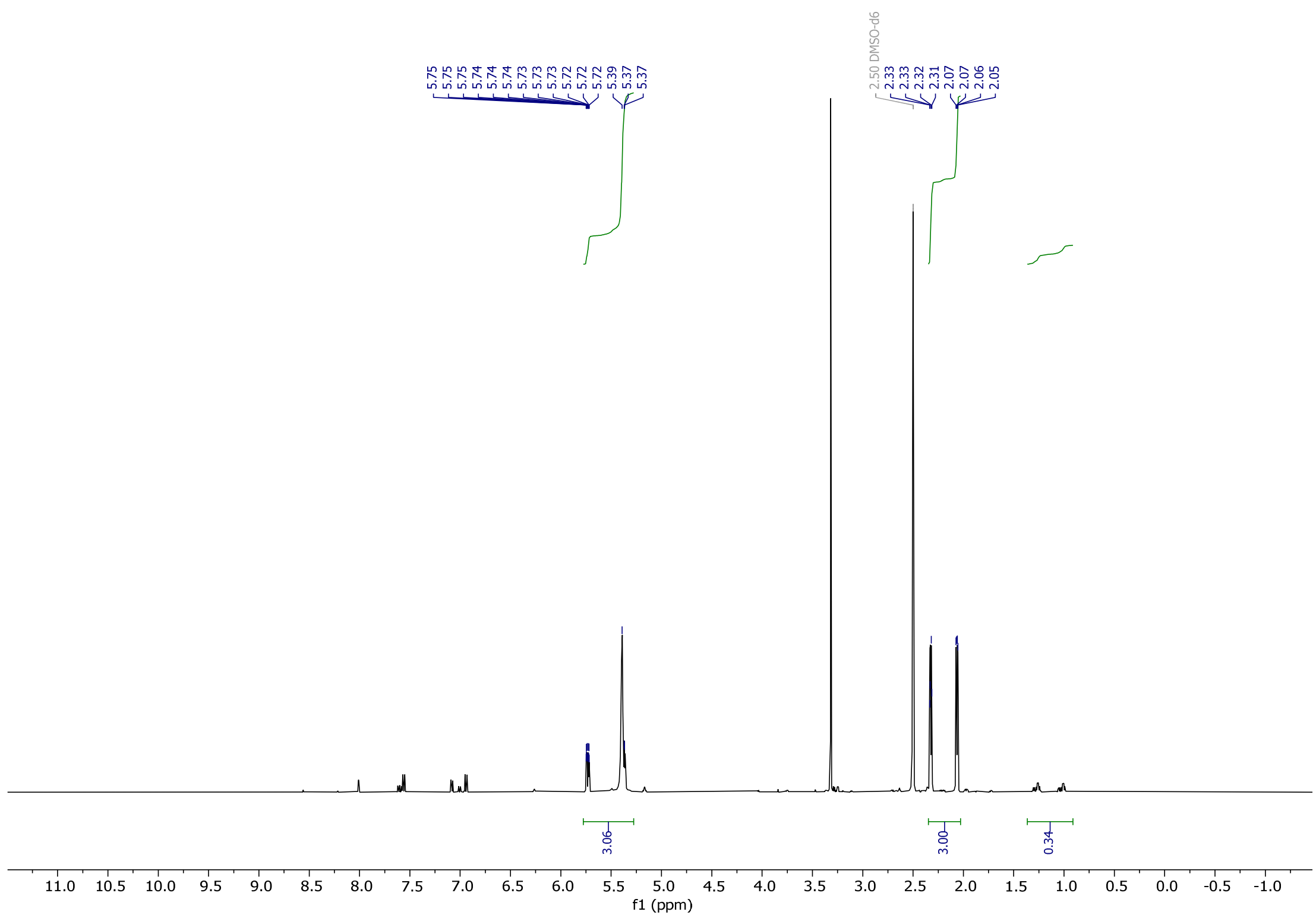

^{13}C-NMR (101 MHz, DMSO-d$_6$):

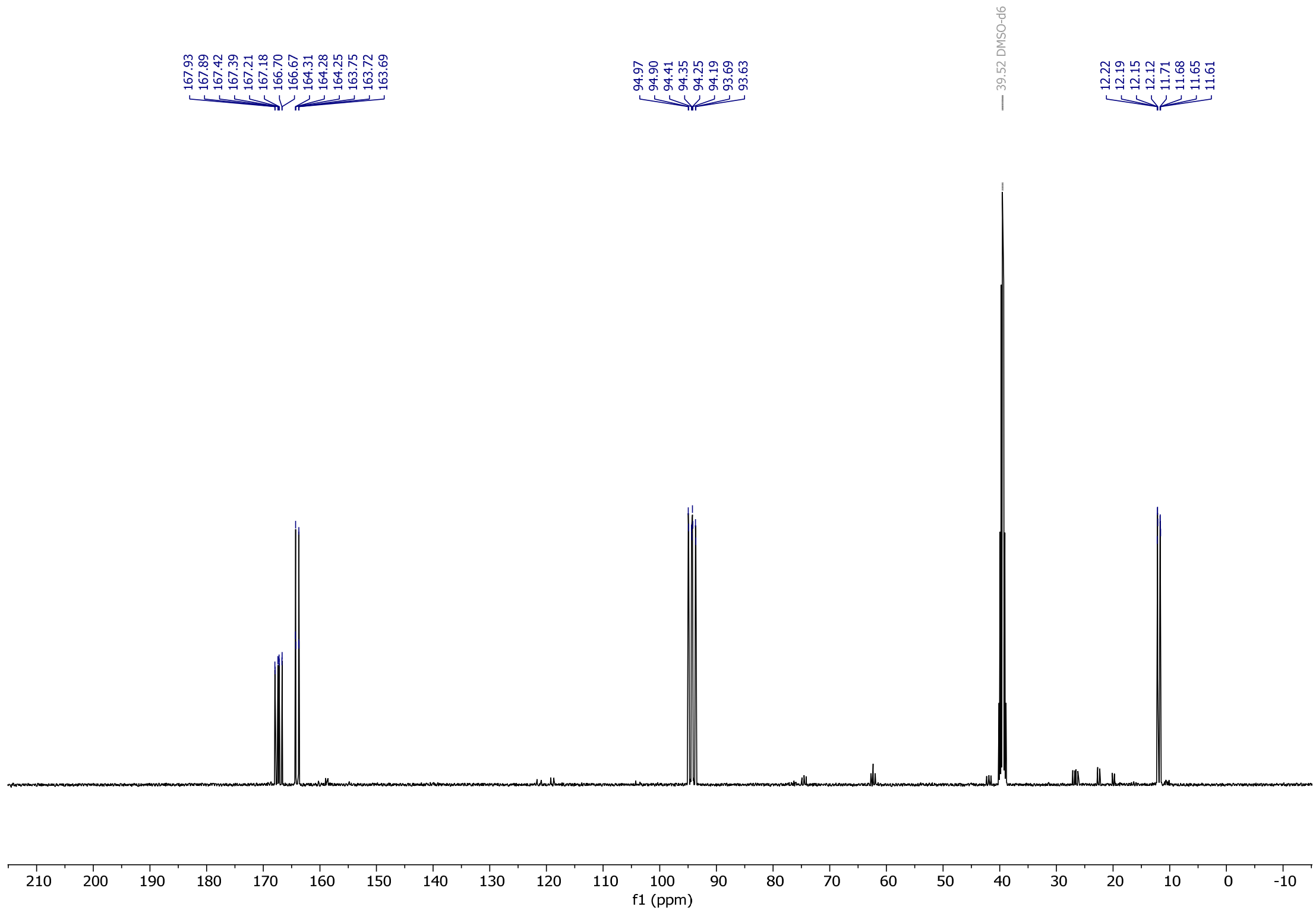

4-Acetamidobenzene-1-sulfonyl chloride-1,2,3,4,5,6-$^{13}C_6$ (4)

^{1}H-NMR (500 MHz, DMSO-d$_6$):

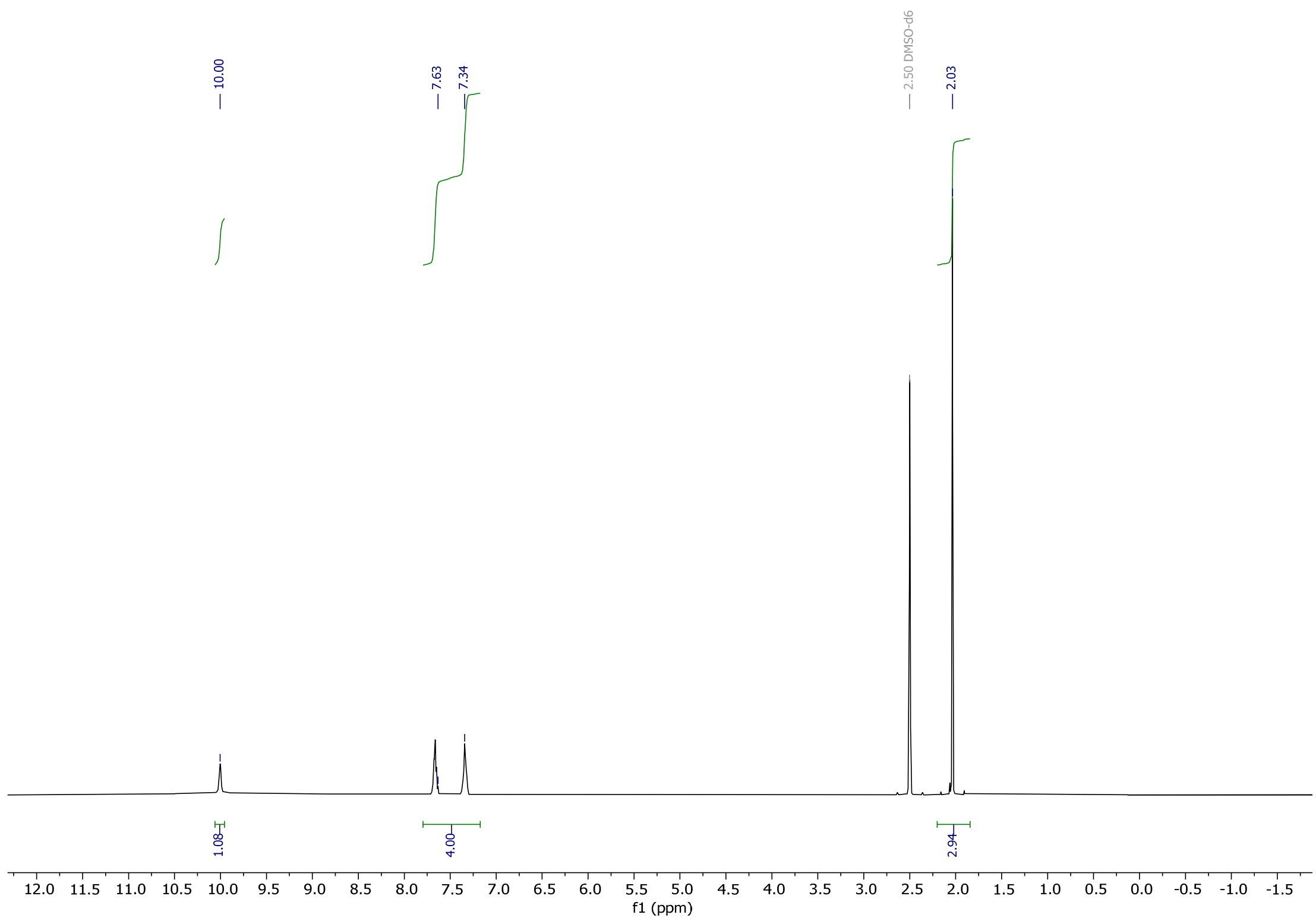

Sulfamethoxazole-1,2,3,4,5,6,7,8,9,10-$^{13}C_{10}$ (5)

^{1}H-NMR (500 MHz, DMSO-d$_6$):

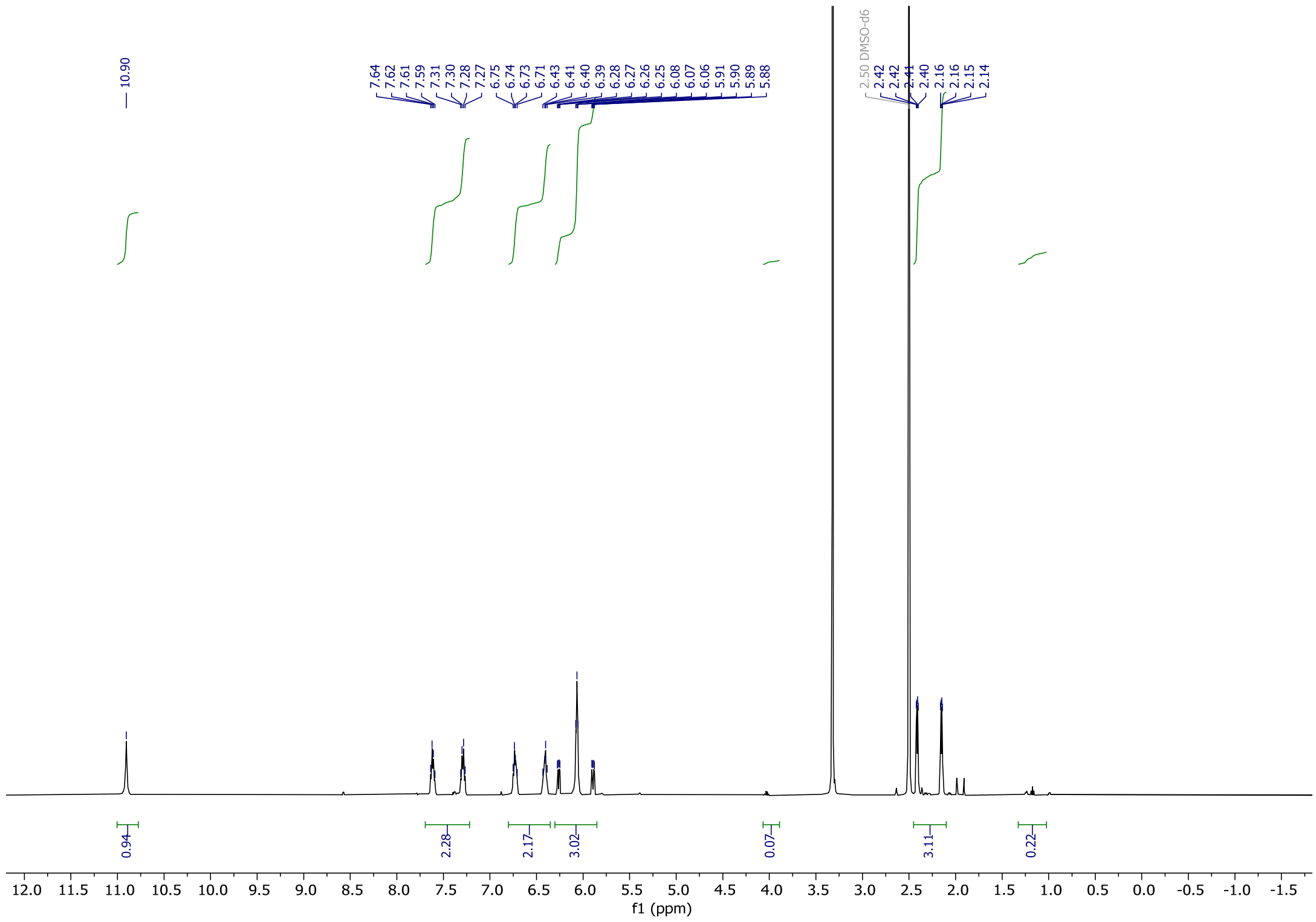

^{13}C-NMR (101 MHz, DMSO-d$_6$):

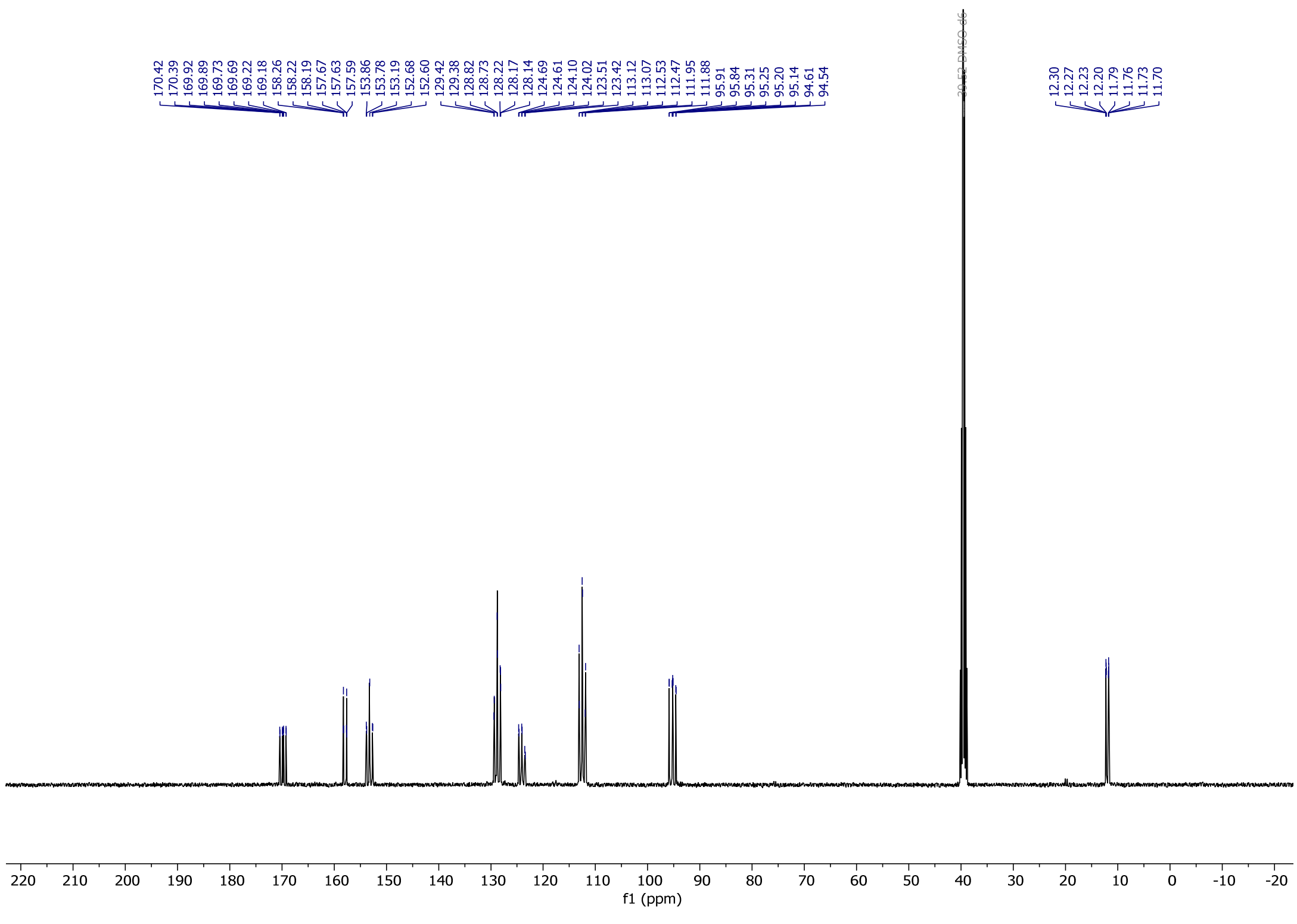

S3 Characterization of the Minerals Used

Specific Surface Area. The specific surface area of the samples was determined by nitrogen adsorption at 77 K with a Quantachrome Autosorb-iQ (Odelzhausen, Germany) according to the Brunauer-Emmet-Teller (BET) theory. Before adsorption measurements, the samples were outgassed and dried under vacuum at 80 °C for 24 h. Adsorption isotherms were measured over a pressure range of 0.05-0.3 bar, with 11 measurement points. The sample weight was adjusted to obtain a total surface area of the sampled material of at least 2 m^2. The goethite used had a specific surface area of 14.6 m^2 g^{-1}.

Crystal Structure. The Powder X-Ray Diffraction PXRD was obtained using a STOE STADI P diffractometer (STOE, Germany) in transmission mode with Mo-K_{a1} radiation (λ = 0.7093 Å, 50 kV, 40 mA), a Ge(111) monochromator, and a Mythen 1K detector. Patterns were collected with an exposure time of 5 s/step between 6° and 48° and a step size of 0.015°/2q. The sample was measured in air-tight sealed capillary (inner ø 0.3 mm, mark tube from special glass, Hilgenberg, Germany). The diffractogram of the MnO_2 particles synthesized showed the reflexes of δ-MnO_2 (Figure S1).[2]

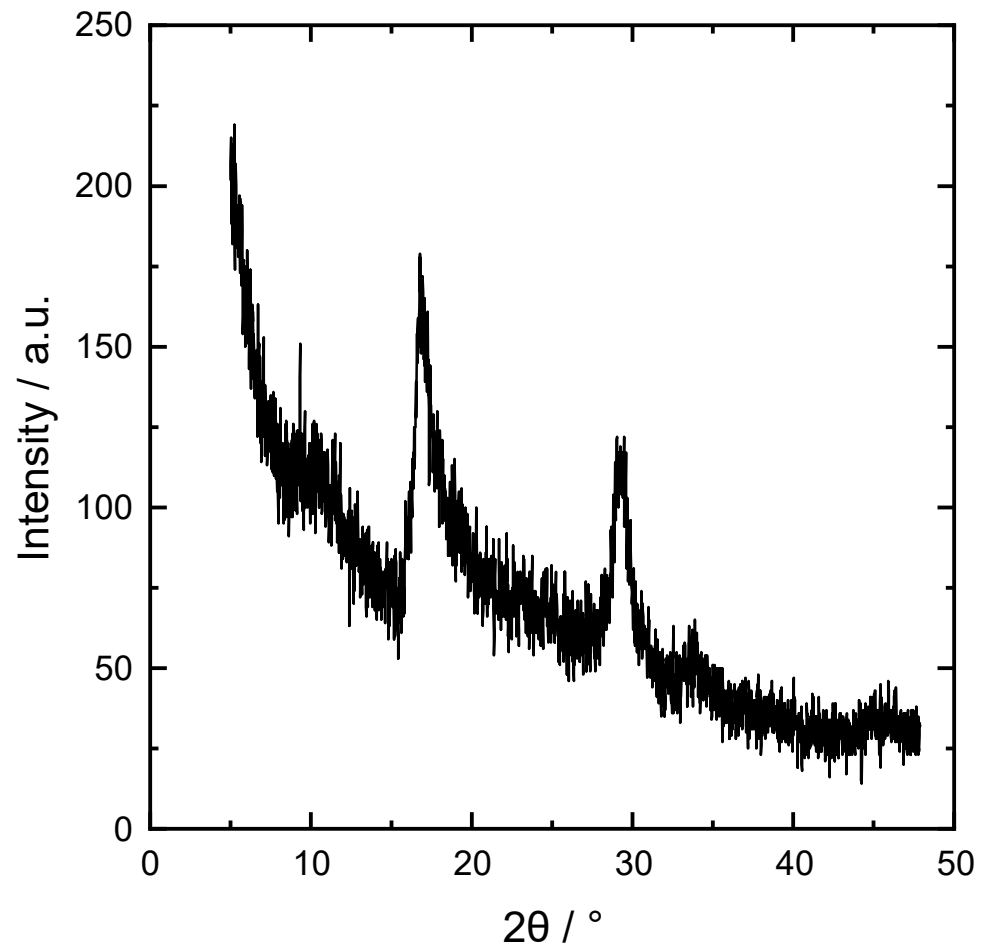

Figure S1: PXRD of the synthesized MnO_2.

S4 Control Experiments for the Transformation Reactions of Sulfamethoxazole

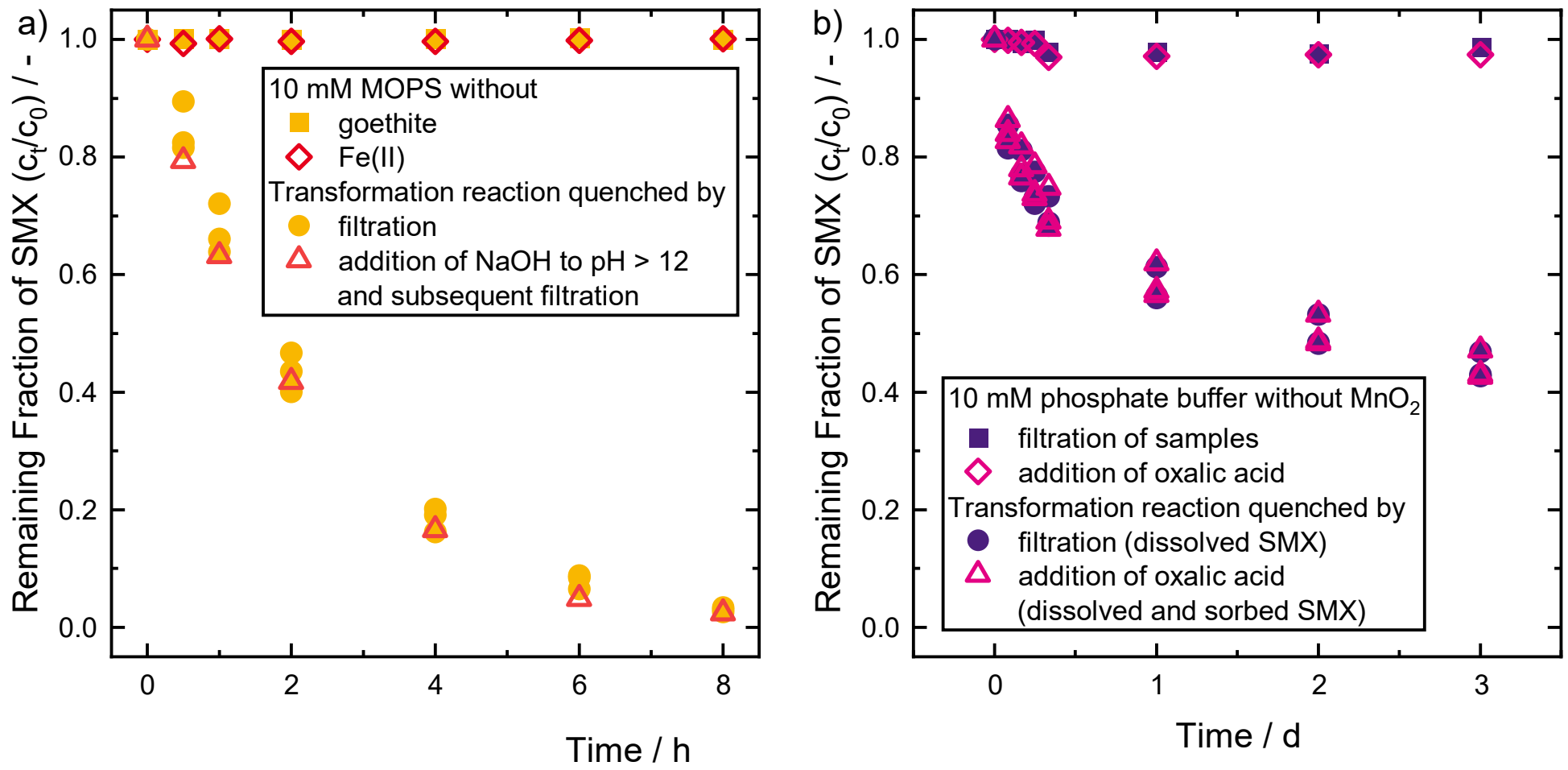

Figure S2: Control experiments for the transformation of SMX, a) by goethite and Fe(II) (10 mM MOPS at pH 7.0 ± 0.2, 0.2 M NaCl, 50 µM SMX) either with Fe(II) (0.5 mM) or goethite (8 g L^{-1}) comparing different quenching methods by either direct filtration or addition of NaOH and subsequent filtration, and b) by MnO_2 (10 mM phosphate buffer at pH 3.0 ± 0.2, 20 µM SMX) either without MnO_2 or with 69 g L^{-1} MnO_2 (dried after synthesis) comparing the amount of dissolved and sorbed SMX during the transformation.

S5 GC-IRMS Measurements

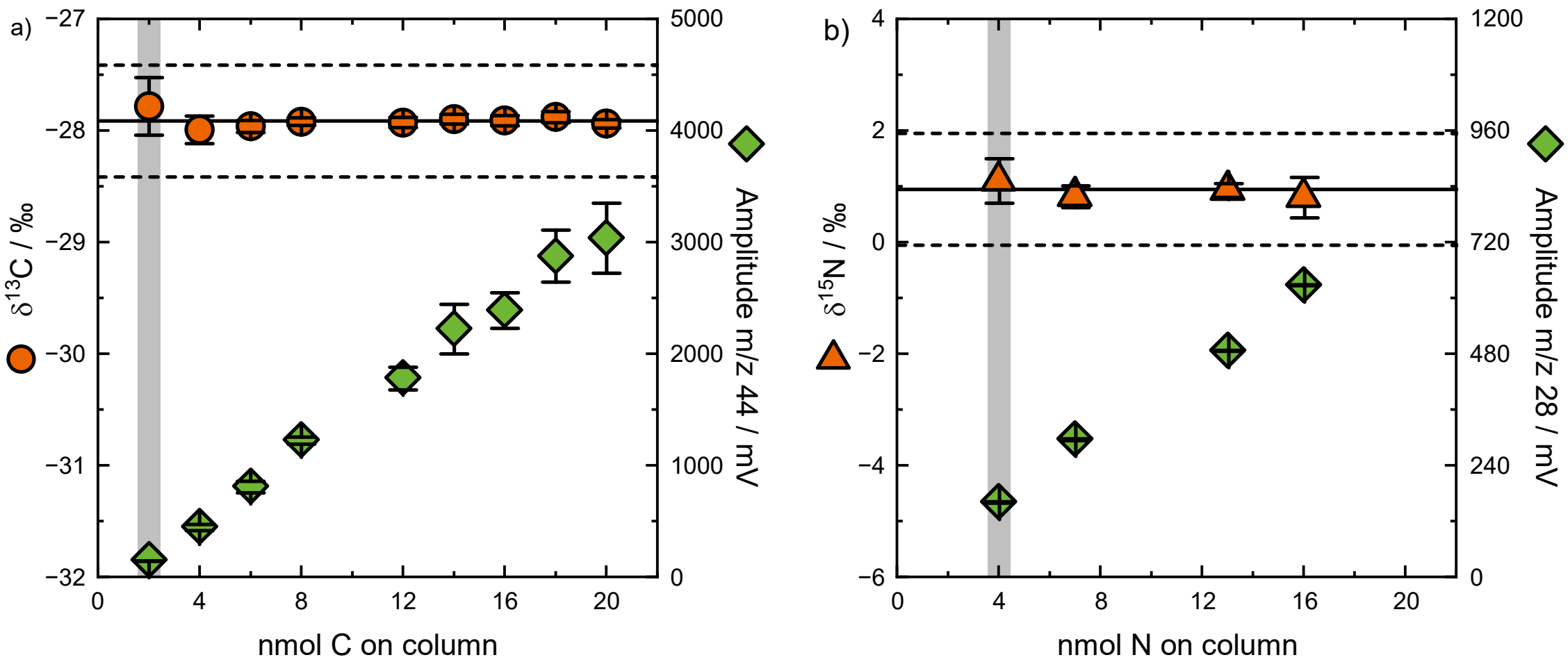

Figure S3: Amount-dependency of accurate a) carbon and b) nitrogen isotope analysis using the in-house SMX standard by GC-IRMS, showing the mean $\delta^{13}C$ or $\delta^{15}N$ values in per mil and the respective peak amplitudes of the main mass trace m/z 44 or m/z 28 in mV of each measured concentration; error bars of both parameters reflect standard deviations (±σ) of triplicate measurements, the grey bars indicate method quantification limits according to the moving mean procedure reported by Jochmann et al.[3]; calculated moving means are represented by solid lines encompassed by a ± 0.5‰ ($\delta^{13}C$) or ± 1‰ ($\delta^{15}N$) interval.

S6 Details on the LC-ESI-Orbitrap Method

Table S2: Ion source settings, scan properties, and MS^2 scan properties of the LC-ESI-Orbitrap method used for TP analysis.

Ion Source Settings	
Spray voltage	Static
Positive ion (V)	3400
Negative ion (V)	2300
Gas mode	Static
Sheath gas (Arb)	40
Aux gas (arb)	8
Sweep gas (arb)	1
Ion transfer tube (°C)	300
Vaporizer temp (°C)	275
Scan Properties	
Orbitrap resolution	120k
Scan range (m/z)	67-1000
RF lens (%)	70
AGC target	Standard
Maximum injection time Mode	Auto
Microscans	1
Polarity	Positive or Negative
MS^2 Scan Properties	
Isolation window (m/z)	1.5
Isolation offset	Off
Collision Energy Type	Normalized
HCD Collision energies (%)	30, 60, 90 or 15, 45, 65
Orbitrap resolution	30K
Scan range (m/z)	Auto
AGC target	Standard
Maximum injection time Mode	Auto
Microscans	1

S7 Rayleigh-Plots

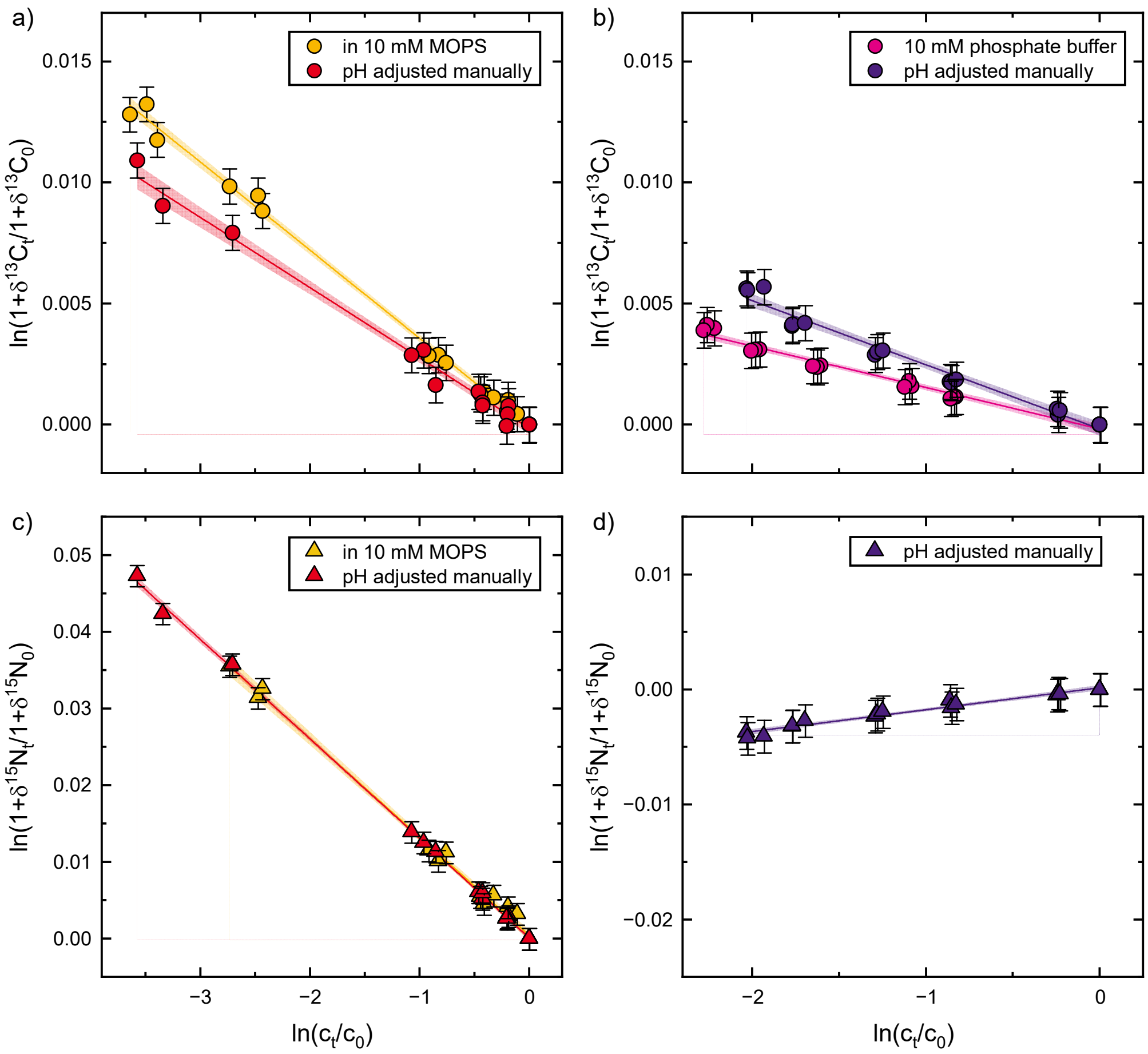

Figure S4: Rayleigh plots with regressions and 95% confidence intervals from the transformation of SMX by Fe(II)-amended goethite suspensions in the absence and presence of MOPS buffer at pH 7.0 ± 0.2 for a) carbon and c) nitrogen isotope analysis; and by MnO_2 suspensions in the absence and presence of phosphate buffer at pH 3.0 ± 0.2 for b) carbon and d) nitrogen isotope analysis.

S8 Transformation Products Formed during the Reductive Transformation of Sulfamethoxazole by Fe(II)-Amended Goethite Suspensions

Table S3: Transformation products detected during the reductive transformation of SMX by Fe(II)-amended goethite suspensions, with their name (TP followed by the molecular mass of the proposed neutral structure), retention time (t_R), measured m/z as $[M+H]^+$, derived elemental composition, calculated m/z as $[M+H]^+$, and the deviation from measured to calculated m/z; only peaks with a peak area ratio relative to the initial SMX peak greater than 1% at λ = 268 nm were reported, noting that the peak at t_R = 15.5 min in the UV spectrum was not detected during the LC-ESI-Orbitrap using positive ionization.

Name	t_R / min	Measured m/z $[M+H]^+$	Elemental Composition [M+H]	Calculated m/z	Deviation / ppm
Sulfanilamide	6.6	173.0378	$C_6H_9O_2N_2S$	173.0379	-0.47
TP255	12.4	256.0748	$C_{10}H_{14}O_3N_3S$	256.0750	-1.07
TP271	13.9	272.0699	$C_{10}H_{14}O_4N_3S$	272.0700	-0.03
TP253	15.7	254.0591	$C_{10}H_{12}O_3N_3S$	254.0594	-1.06
SMX	18.6	254.0592	$C_{10}H_{12}O_3N_3S$	254.0594	-0.76

Table S4: The suggested structures of the most abundant transformation products detected during the reductive transformation of SMX by Fe(II)-amended goethite suspensions, with their name (TP followed by the molecular mass of the proposed neutral structure), calculated molecular mass of the suggested neutral structure, m/z of fragments from MS/MS experiments, suggested structure, and information about structure verification by an authentic standard.

Name	Calculated molecular mass of neutral suggested structure / g mol^{-1}	Fragments from MS/MS experiment m/z	Suggested Structure	Identification Confidence according to Schymanski et al.[4]
Sulfanilamide	172.0307	156, 108, 92, 65	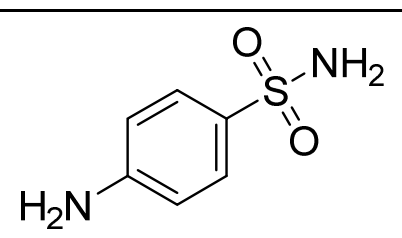	Level 1
TP255	255.0678	214, 156, 108, 101, 92, 65	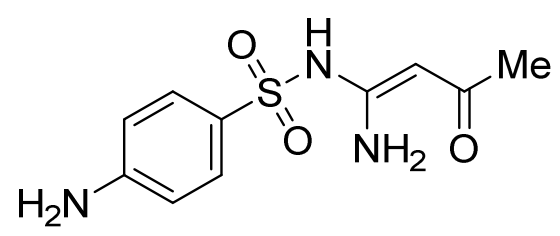	Level 3
TP271	271.0627	254, 173, 156, 108, 92, 65	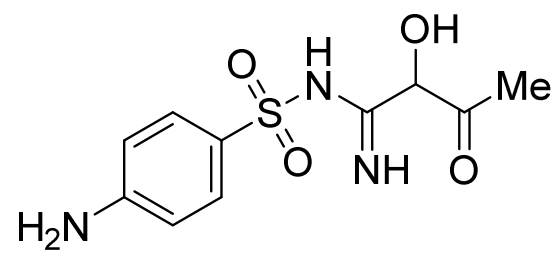	Level 3
TP253	253.0521	156, 140, 108, 99, 92, 65	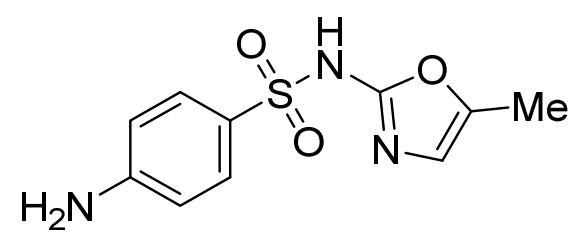	Level 3
SMX	253.0521	156, 108, 99, 92, 65	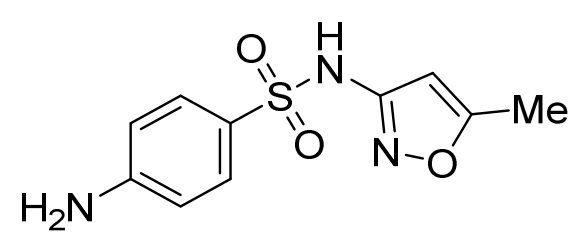	Level 1

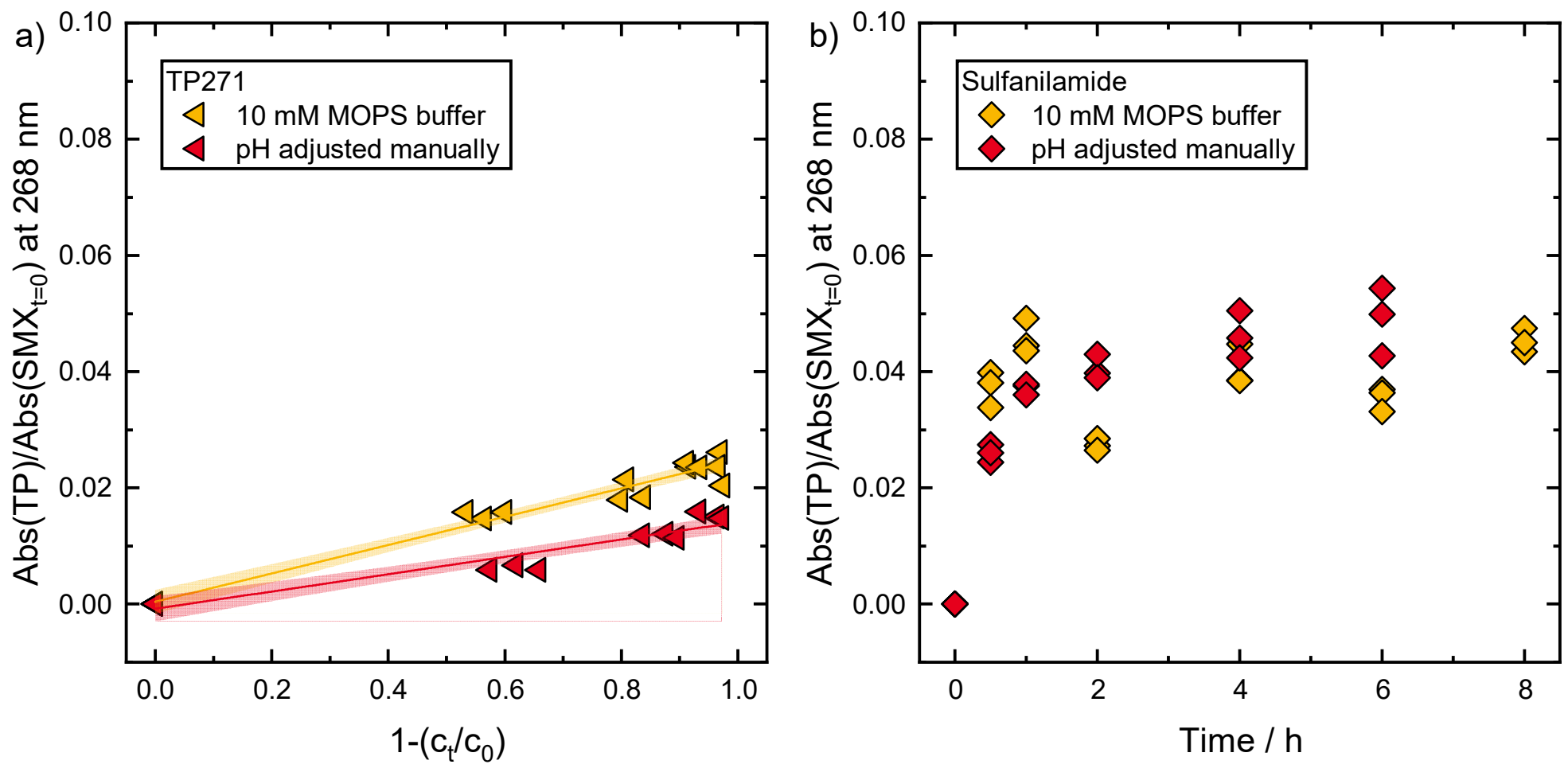

Figure S5: Formation of the minor transformation products a) TP271 and b) sulfanilamide detected during the reductive transformation of SMX by Fe(II)-amended goethite suspensions using a 10 mM MOPS buffer and adjusting the pH manually through the experiment at pH 7.0 ± 0.2.

S9 Reaction Kinetics of the Transformation of Sulfamethoxazole by Fe(II)-Amended Goethite Suspensions

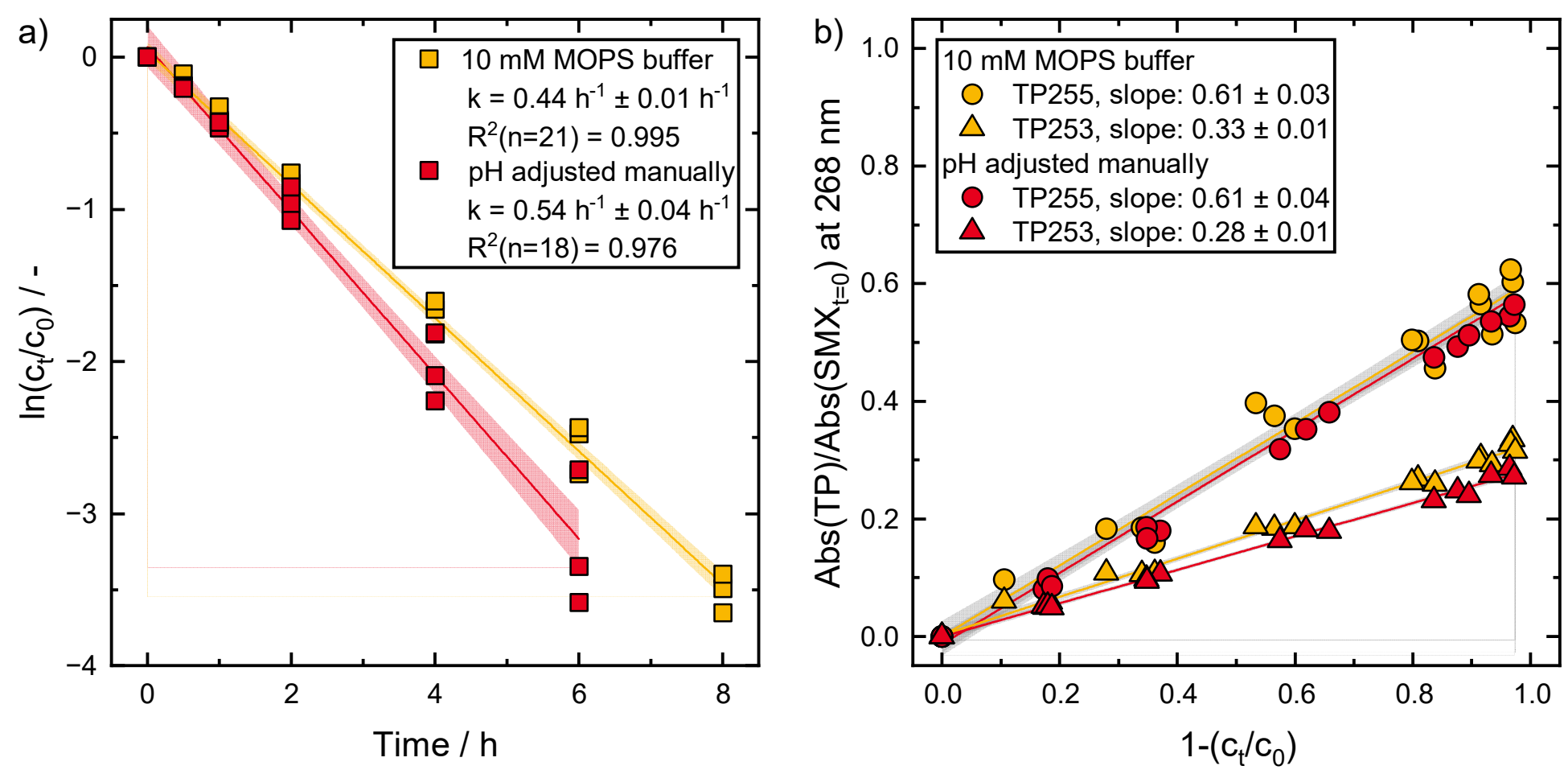

Figure S6: a) First-order reaction kinetics from the transformation of SMX by Fe(II)-amended goethite suspensions in the absence and presence of MOPS buffer at pH 7.0 ± 0.2, and b) Relative product yields as a result from the slope from the peak area ratios (peak area of TP at a certain time point over the initial SMX peak area) at the wavelength of 268 nm vs the consumed fraction of SMX; lines in the respective color represent the regressions together with their 95% CIs in grey.

S10 Transformation Products Formed during the Oxidative Transformation of Sulfamethoxazole by MnO_2 Suspensions

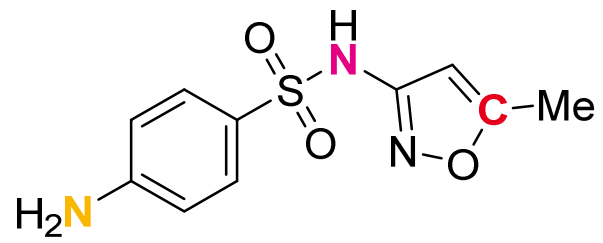

Scheme S2: Structure of sulfamethoxazole, indicating the respective positions, which have been labeled with a ^{13}C or ^{15}N, respectively; the colors are further used to indicate the respective atoms in the proposed structures of TPs in Tables S5 and S6.

Table S5: Transformation products detected during the oxidative transformation of SMX by MnO_2 suspensions in phosphate buffer, with their name (TP followed by the molecular mass of the proposed neutral structure and the molecular mass of the ^{13}C-labeled proposed neutral structure), retention time (t_R), the number of carbon atoms derived from the experiment performed with $^{13}C_{10}$-SMX, measured m/z as $[M-H]^-$, derived elemental composition, calculated m/z as $[M-H]^-$, the deviation from measured to calculated m/z, the determined number of carbon or nitrogen atoms determined from a transformation experiment performed with the respective labeled SMX, m/z of fragments from MS^2 experiments (with the number of carbon atoms and information on the amount of specific carbon and nitrogen atoms in brackets), suggested structure, with if applicable the position of the respective carbon and nitrogen atoms, and information about structure verification by an authentic standard.

Name	t_R / min	Number of Carbon Atoms	Measured m/z $[M+H]^-$	Elemental Composition [M+H]	Calculated m/z	Deviation / ppm	# N	# N	# C	Fragments from MS^2 as m/z	Suggested Structure	Identification Confidence according to Schymanski et al.[4]
TP260 (TP268)	10.1	8	259.0033	$C_8H_7O_6N_2S$	259.0019	4.79	0	1	1	153 ($C_1C_5N_1$), 99 (C_4), 71 (C_3), 64	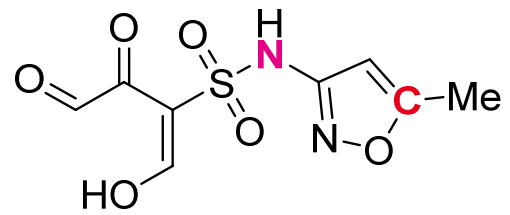	Level 3
TP176 (TP181)	12.7	5	175.0185	$C_5H_7O_3N_2S$	175.0172	1.31	0	1	1	79 (C_1), 64	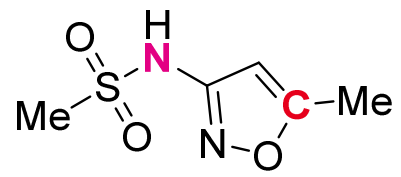	Level 3
TP218 (TP225)	16.3	7	217.0290	$C_7H_9O_4N_2S$	217.0278	1.29	0	1	1	138 ($C_1C_5N_1$), 121 (C_3), 57 (C_3)	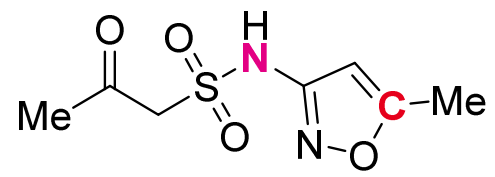	Level 3

(Table S5 continued on the next page)

TP325 (TP337)	17.5	12	324.0299	$C_{12}H_{10}O_6N_3S$	324.0285	1.44	1	1	1	252 ($C_1C_9N_1N_1$), 159 ($C_1C_4N_1N_1$), 146 ($C_6N_1N_1$), 107 (C_6N_1), 96 ($C_1C_3N_1$), 91 (C_6N_1), 82 (C_1C_3),	-	Level 5
TP483 (TP499)	18.4	16	482.0082	$C_{16}H_{12}O_9N_5S_2$	482.0071	1.15	1	2	1	411 ($C_1C_{13}N_1N_1$), 346 ($C_1C_{13}N_1N_2$), 250 ($C_{10}N_1N_1$), 185 ($C_{10}N_1N_1$)	-	Level 5
SMX	18.8	10	254.0590	$C_{10}H_{12}O_3N_3S$	252.0450	-1.28	1	1	1	-	-	Level 1
TP281 (TP292)	19.4	11	280.0400	$C_{11}H_{10}O_4N_3S$	280.0387	1.30	1	1	1	184 (C_7N_1), 120 (C_7N_1), 92 (C_6N_1)	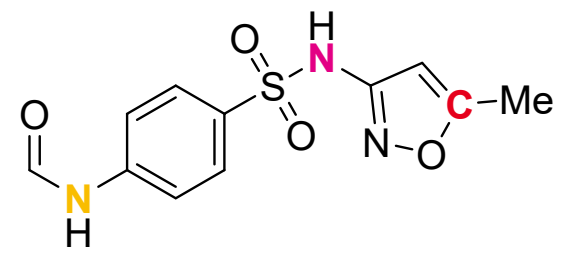	Level 3
TP295 (TP306)	20.2	11	294.0193	$C_{11}H_8O_5N_3S$	294.0179	1.40	1	1	1	198 (C_7N_1), 134 (C_7N_1), 133 (C_7N_1),	-	Level 5
TP509 (TP527)	22.1	18	508.0240	$C_{18}H_{14}O_9N_5S_2$	508.0227	1.21	1	2	2	348 ($C_1C_{13}N_1N_1$), 276 ($C_1C_{12}N_1N_1$), 182 (C_7N_1), 115 (C_8N_1), 82 ($C_1C_3N_1$)	-	Level 5
TP298 (TP308)	22.5	10	297.0302	$C_{10}H_9O_5N_4S$	297.0288	1.36	1	1	1	201 (C_7N_1), 137 (C_6N_1), 107 (C_6N_1)	-	Level 5

(Table S5 continued on the next page)

TP455 (TP471)	23.5	16	454.0495	$C_{16}H_{16}O_7N_5S_2$	454.0486	0.96	1	2	2	278 ($C_1C_{10}N_1N_1$), 252 ($C_1C_9N_1N_1$), 182 (C_7N_1), 160 ($C_1C_3N_1$), 118 (C_7N_1), 92 (C_6N_1), 79 (C_1), 64	-	Level 5
TP504 (TP524)	25.0	20	503.0815	$C_{20}H_{19}O_6N_6S_2$	503.0802	1.28	2	2	2	407 ($C_1C_{15}N_2N_1$), 311 ($C_{12}N_2$), 247 ($C_{12}N_2$)	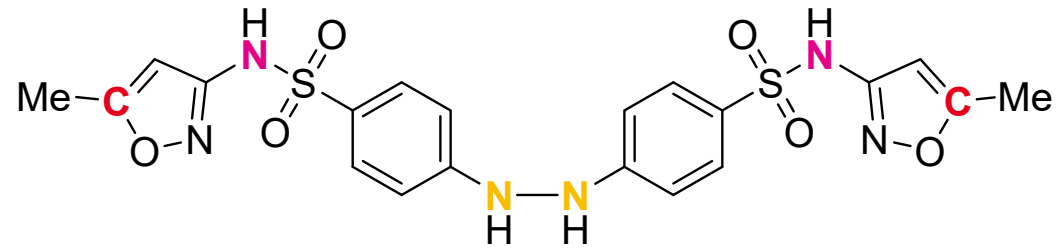	Level 3
TP283 (TP293)	26.8	10	282.0193	$C_{10}H_8O_5N_3S$	282.0179	1.30	1	1	1	186 (C_6N_1), 138 (C_6N_1), 122 (C_6N_1)	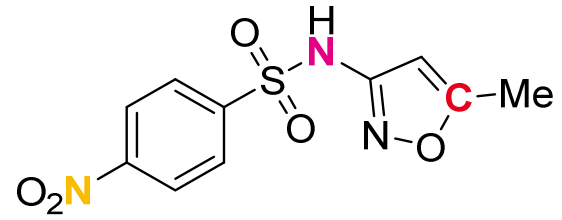	Level 3
TP500 (TP520)	35.1	20	499.0501	$C_{20}H_{15}O_6N_6S_2$	499.0489	1.12	2	2	2	355 ($C_1C_{15}N_2N_1$), 194 ($C_{12}N_2$)	-	Level 5

Table S6: Transformation products detected during the oxidative transformation of SMX by MnO_2 suspensions in phosphate buffer, with their name (TP followed by the molecular mass of the proposed neutral structure and the molecular mass of the ^{13}C-labeled proposed neutral structure), retention time (t_R), the number of carbon atoms derived from the experiment performed with $^{13}C_{10}$-SMX, measured m/z as $[M+H]^+$, derived elemental composition, calculated m/z as $[M+H]^+$, the deviation from measured to calculated m/z, the determined number of carbon or nitrogen atoms determined from a transformation experiment performed with the respective labeled SMX, m/z of fragments from MS^2 experiments (with the number of carbon atoms and information on the amount of specific carbon and nitrogen atoms in brackets), suggested structure, with if applicable the position of the respective carbon and nitrogen atoms, and information about structure verification by an authentic standard.

Name	t_R / min	# C total	Measured m/z $[M+H]^+$	Elemental Composition [M+H]	Calculated m/z	Deviation / ppm	# N	# N	# C	Fragments from MS/MS experiment m/z	Suggested Structure	Identification Confidence according to Schymanski et al.[4]
3-Amino-5-methyl isoxazole	9.2	4	99.0552	$C_4H_7ON_2$	99.0553	-1.30	0	1	1	72 (C_1C_2), 44 (C_2)	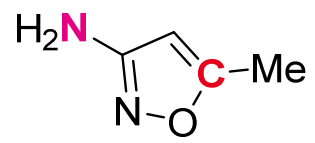	Level 1
TP220 (TP226)	12.7	6	221.0226	$C_6H_9O_5N_2S$	221.0227	-0.13	0	1	1	203 ($C_1C_5N_1$), 161($C_1C_3N_1$), 125 ($C_1C_4N_1$), 111 ($C_1C_3N_1$) 97 ($C_1C_3N_1$)	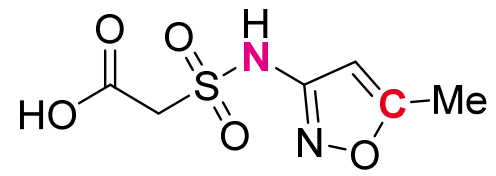	Level 3
TP295 (TP306)	14.9	11	296.0811	$C_{11}H_{14}O_3N_5S$	296.0812	-0.23	1	2	1	279 ($C_1C_{10}N_1N_1$), 135 ($C_7N_1N_1$), 118 ($C_7N_1N_1$)	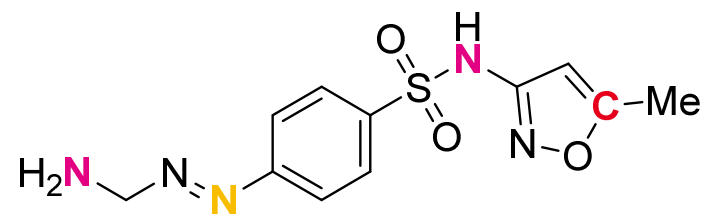	Level 3
TP240 (TP247)	16.3	7	241.0254	-	-	-	0	1	1	143 (C_3), 121 ($C_1C_3N_1$)	-	Level 5
TP325 (TP337)	17.5	12	326.0439	$C_{12}H_{12}O_6N_3S$	326.0441	-0.58	1	1	1	282 ($C_1C_{10}N_1N_1$), 184 (C_7N_1), 148 (C_7N_1), 120 (C_7N_1)	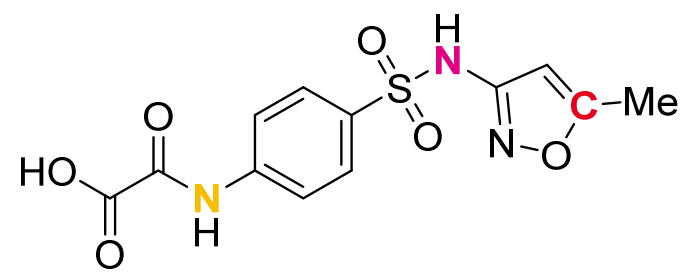	Level 3
SMX	18.8	10	254.0591	$C_{10}H_{12}O_3N_3S$	254.0594	-1.20	1	1	1	-	-	Level 1

S11 GC-IRMS Measurement for Qualitative Detection of $^{13}CO_2$ during the Oxidative Transformation of Sulfamethoxazole

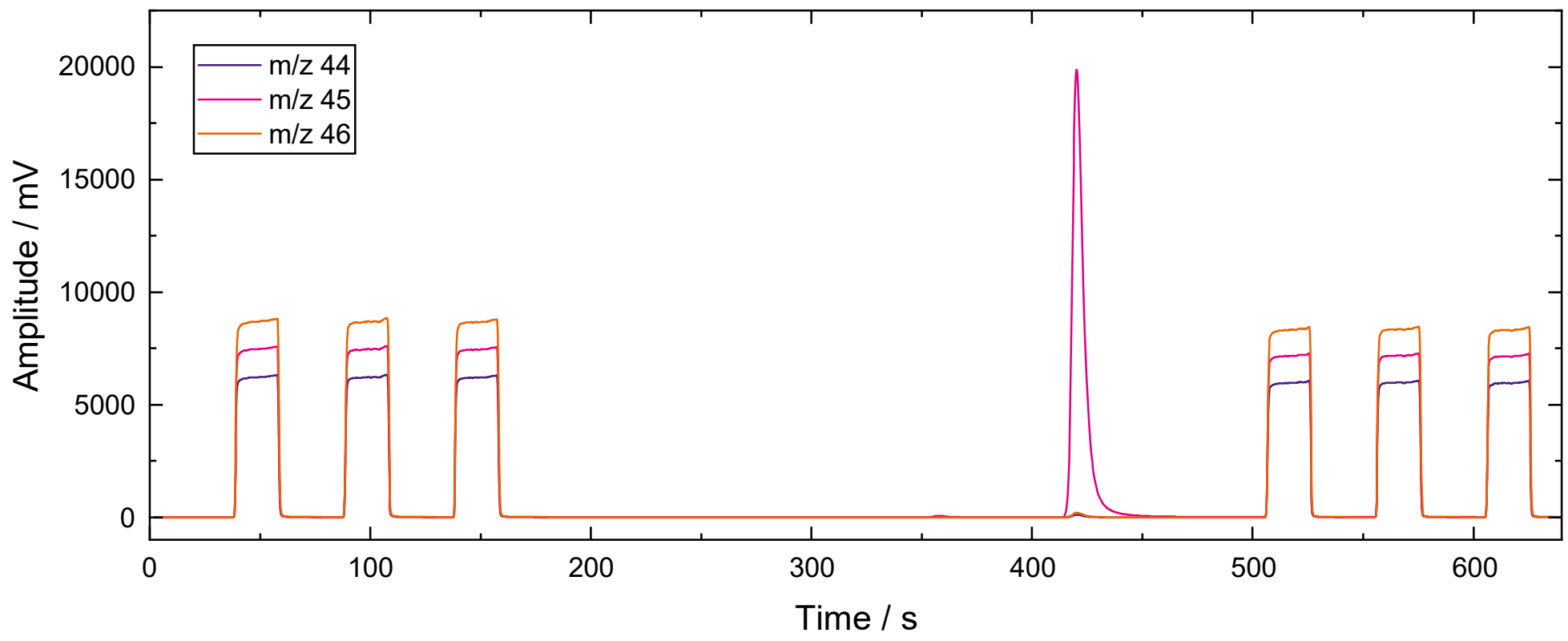

Figure S7: GC-IRMS chromatogram of the headspace injection from a MnO_2-mediated SMX transformation after 14 days.

S12 Transformation Kinetics of Sulfamethoxazole by MnO_2 Suspensions

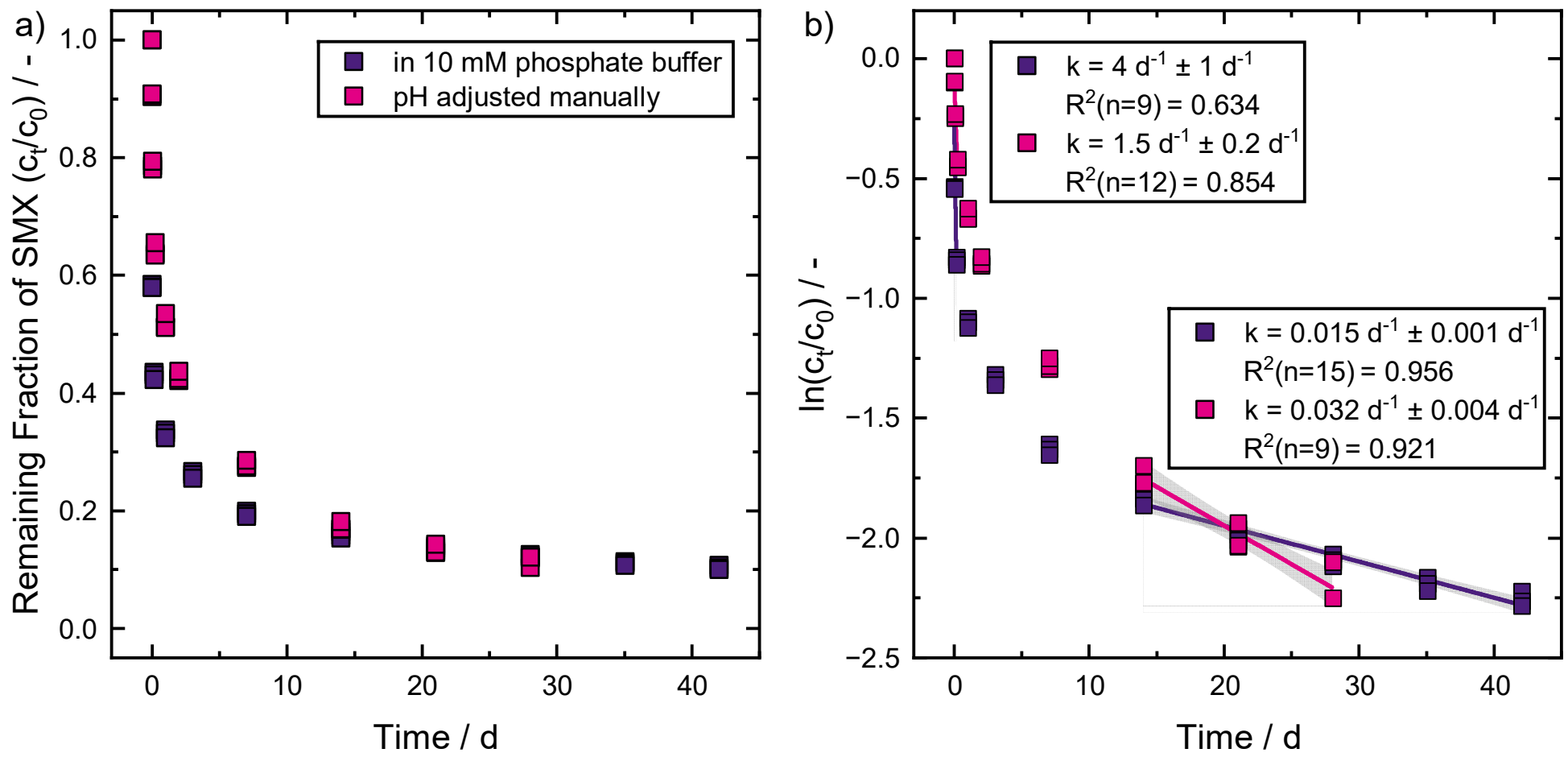

Figure S8: a) Time course for the abiotic transformation of SMX in aqueous MnO_2 suspensions in a 10 mM phosphate buffer and in the absence of a buffer, with manual pH adjustment throughout the experiments, both at pH 3.0 ± 0.2, b) first-order reaction rate constants determined at the beginning and end of the reaction.

S13 References

(1) Canavan, A.; Dirr, C.; Elsner, M. Intramolecularly Labeled Reference Standards of Sulfamethoxazole for Fragment-Specific Isotope Analysis by Electrospray Ionization Orbitrap Mass Spectrometry. *J. Am. Soc. Mass. Spectrom.* **2026**, *37* (3), 717–726.

(2) Villalobos, M.; Toner, B.; Bargar, J.; Sposito, G. Characterization of the manganese oxide produced by pseudomonas putida strain MnB1. *Geochim. Cosmochim. Acta* **2003**, *67* (14), 2649–2662.

(3) Jochmann, M. A.; Blessing, M.; Haderlein, S. B.; Schmidt, T. C. A new approach to determine method detection limits for compound-specific isotope analysis of volatile organic compounds. *Rapid Commun. Mass Spectrom.* **2006**, *20* (24), 3639–3648.

(4) Schymanski, E. L.; Jeon, J.; Gulde, R.; Fenner, K.; Ruff, M.; Singer, H. P.; Hollender, J. Identifying Small Molecules via High Resolution Mass Spectrometry: Communicating Confidence. *Environ. Sci. Technol.* **2014**, *48* (4), 2097–2098.
